# Innovative mitochondria-based holistic 3PM approach to female health status: Facts and outlook

**DOI:** 10.1007/s13167-026-00438-7

**Published:** 2026-02-11

**Authors:** Martin Pesta, Vadim Goncharenko, Vlastimil Kulda, Jiri Polivka, Jasur Rizaev, Olga Golubnitschaja

**Affiliations:** 1 Department of Biology, Faculty of Medicine in Pilsen, Charles University, Pilsen, Czech Republic; 2 Department of Immunochemical Diagnostics, University Hospital Pilsen, Pilsen, Czech Republic; 3 Gynecologic Department, Clinical Hospital “Pheophania”, Kyiv, Ukraine; 4 Department of Medical Chemistry and Biochemistry, Faculty of Medicine in Pilsen, Charles University, Pilsen, Czech Republic; 5 Department of Histology and Embryology, Faculty of Medicine in Pilsen, Charles University, Pilsen, Czech Republic; 6 Department of Histology and Embryology, Second Faculty of Medicine, Charles University, Prague, Czech Republic; 7 Samarkand State Medical University, Samarkand, Uzbekistan; 8 Predictive, Preventive and Personalised (3P) Medicine, Department of Radiation Oncology, University Hospital Bonn, Rheinische Friedrich-Wilhelms- Universität Bonn, 53127 Bonn, Germany

**Keywords:** Predictive preventive personalised medicine (PPPM / 3PM), Life quality, Mitochondrial health, Homeostasis, Health risk assessment, Patient phenotyping and stratification, Flammer syndrome phenotype, Sympathetic overdrive, Chronic fatigue, Chronic inflammation, Hormonal stress, Pregnancy, Gestational diabetes, Female cancers, Individualised protection against health-to-disease transition, Individualised rehabilitation programme, Patient-friendly non-invasive approach, Tear fluid analysis, AI, Expert recommendations

## Abstract

Throughout a woman’s life, energy metabolism faces more volatile demands compared to men, driven by major hormonal transitions like puberty, the menstrual cycle, pregnancy, and menopause. Mitochondria, as central organelles for energy production and vital cellular biosensors, are consequently subjected to substantial physiological stress. When the body’s compensatory mechanisms are overwhelmed, particularly in states of suboptimal health, compromised mitochondrial functionality is characterised by increased generation of reactive oxygen species (ROS) frequently associated with a chronification of the low-grade inflammation and development of follow-up pathologies. Compromised health and shifted homeostasis of mitochondria are crucial for development and progression of a broad spectrum of disorders in female subpopulations which can be exemplified by chronic fatigue, gestational diabetes mellitus, preeclampsia, polycystic ovary syndrome, and gynaecologic cancers, amongst others. This article strongly advocates for the implementation of an innovative, mitochondria-based holistic approach within the framework of Predictive, Preventive, and Personalised Medicine (3PM). 3PM-guided strategy focuses on the early detection of reversible health damage shifting care from reactive treatment to individualised proactive, risk-adapted management of the health conditions. The integration and interpretation of the multimodal data, supported by artificial intelligence, enable the stratification of individuals in suboptimal health conditions and affected patients for cost-effective protective measures tailored to individualised patient profiles, therapeutic interventions, and personalised pre- and rehabilitation programmes. In summary, by utilising mitochondrial biosensorics for monitoring systemic effects in a holistic manner, the presented 3PM-guided innovation offers a robust model for protecting individuals against health-to-disease transition and disease progression in affected patient cohorts.

## Preamble

Throughout a woman’s life, energy metabolism is subject to significantly higher demands in terms of changes that of men. Sexual dimorphism between women and men, arising from genetic differences and subsequently shaped by hormonal influences, places substantial demand on female metabolic regulation and drives a range of physiological adaptations, particularly those associated with healthy physiologic development from childhood to adulthood as well as with partnership and motherhood with follow-up [1]. Major hormonal transitions during puberty, the regular menstrual cycle, pregnancy, the menopausal transition, and menopause itself impose considerable challenges on female energy metabolism, both in terms of physiological stress and the adaptive responses required [2].

**Fig. 1:**
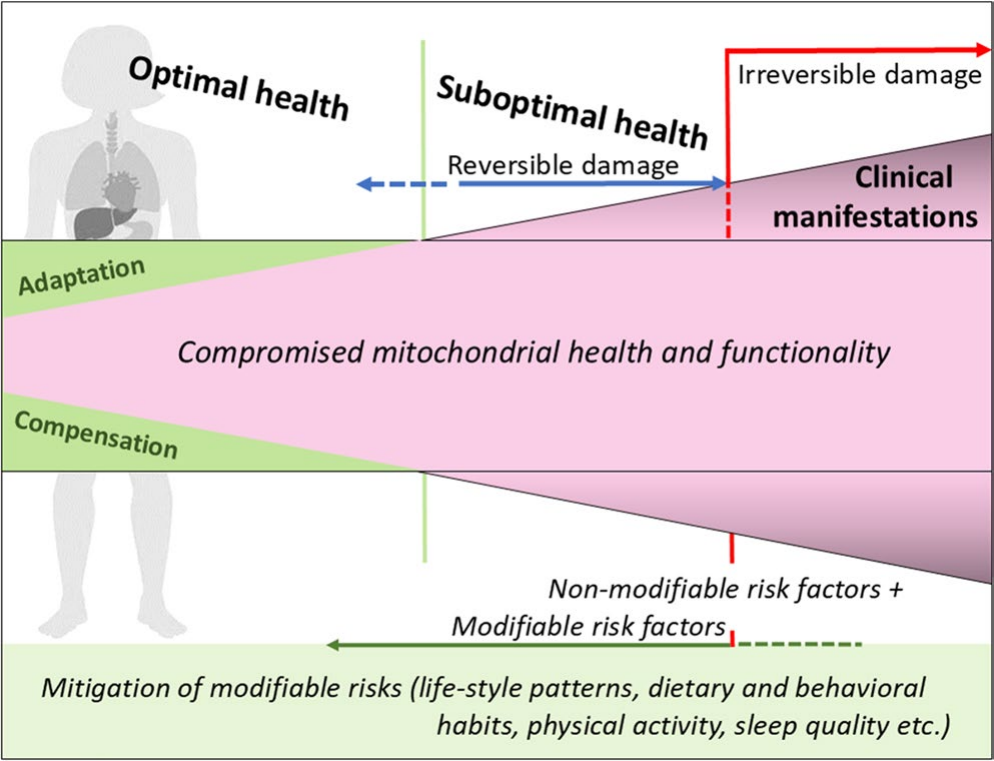
Compensatory capacity of female energy metabolism and mitochondrial health. Under healthy conditions, efficient mitochondrial function supports adaptive hormonal and metabolic regulation. In suboptimal states, impaired mitochondrial health reduces compensatory capacity, promoting oxidative stress and disease risk

These transitions go hand-in-hand with significant changes in the load of mitochondria as energy metabolism central cell organelle and vital biosensors in the human body [Golubnitschaja O. Mitochondrial biosensorics check-up is crucial for physical fitness and exercise intervention quality – Facts and practical recommendations. Clinical Bioenergetics 2025]. This disrupts the optimal functioning of mitochondria with the subsequent ability to compromise their biogenesis, increase the production of oxidative stress and the production of pro-inflammatory factors. Under conditions of optimal health, such effects are typically counter balanced through physiological regulatory mechanisms, including compensation and adaptation processes [3]. However, in cases of illness or suboptimal health (reversible damage to a health condition) or illness (clinically manifested pathology with irreversible damage to a health condition), these compensatory mechanisms may not fully eliminate the adverse effects, which can lead to both short-term and long-term negative consequences as specified in Fig. 1.

At the same time, it should be emphasised that an active approach by women—namely adherence to a healthy lifestyle in combination with the principles of Predictive, Preventive, and Personalised Medicine (3PM)—can substantially mitigate or even prevent these negative consequences of physiological changes. This 3PM approach includes primary care interventions, where reversible damage can be detected early and managed to protect against the transition from health to disease. Secondary care strategies are aimed at protecting against disease progression once pathology is clinically manifested. Both primary and secondary care interventions arise from the understanding of mitochondrial stress followed by compromised cellular homeostasis. These care strategies focus on specifically modifiable factors to reduce preventable risks and restore balance at molecular, sub/cellular and organismal levels [4–10].

The central role of mitochondria arises from the fact that mitochondria regulate a wide range of essential cellular processes, including energy homeostasis, apoptosis, and intracellular signalling. Mitochondria possess crucial roles in cell maintenance, survival and well-being, because they are the main source of energy [11]. Over 90% of ATP, as the main high-energy compound driving many cellular processes, is produced in the mitochondria with the involvement of oxidative decarboxylation of pyruvate, β-oxidation of fatty acids, the Krebs cycle and electron transport chain [12].

Consequently, alterations in mitochondrial function—such as decreased ATP production efficiency, elevated ROS generation, and increased pro-inflammatory cytokine production—can compromise systemic health both acutely and chronically. Some molecules involved in these processes can serve as key biomarkers of mitochondrial health and therapeutic targets [13].

From this perspective, the energy metabolism of women is under strain from puberty onwards, when increasing sex hormone levels affect body composition, promote fat deposition, and alter insulin sensitivity—all of which are key determinants of energy balance [14].

During the menstrual cycle, fluctuations in estrogen and progesterone levels lead to a mild increase in resting energy expenditure (REE) and changes in substrate preference—most notably, an increased reliance on lipid oxidation during the luteal phase) [15–18].

Pregnancy is a period of profound hormonal changes that significantly increase the body’s energy demands, promote the accumulation of energy reserves, and alter nutrient metabolism to meet the needs of the developing fetus [14].

The menopausal transition and menopause are associated with a decline in estrogen and a relative increase in androgens, leading to changes in body composition (increased visceral fat and loss of muscle mass), a reduction in basal metabolic rate, decreased capacity to efficiently utilise free fatty acids, and an elevated risk of insulin resistance and metabolic disorders [3, 19, 20].

Hormonal fluctuations modulate energy metabolism at multiple levels, starting from alterations in taste preferences [17], through energy expenditure and the body’s overall ability to adapt to stress conditions [15], to the extent of oxidative stress production and the modulation of mitochondrial function.

Physiological changes that accompany a woman’s life are also associated with potential pathologies that are linked to these physiological transitions. The most evident examples include conditions related to pregnancy, such as preeclampsia and gestational diabetes mellitus. Given the metabolic nature of these disorders, mitochondrial dysfunction, increased oxidative stress, and elevated production of pro-inflammatory factors represent important components of their pathophysiology.

**Fig. 2:**
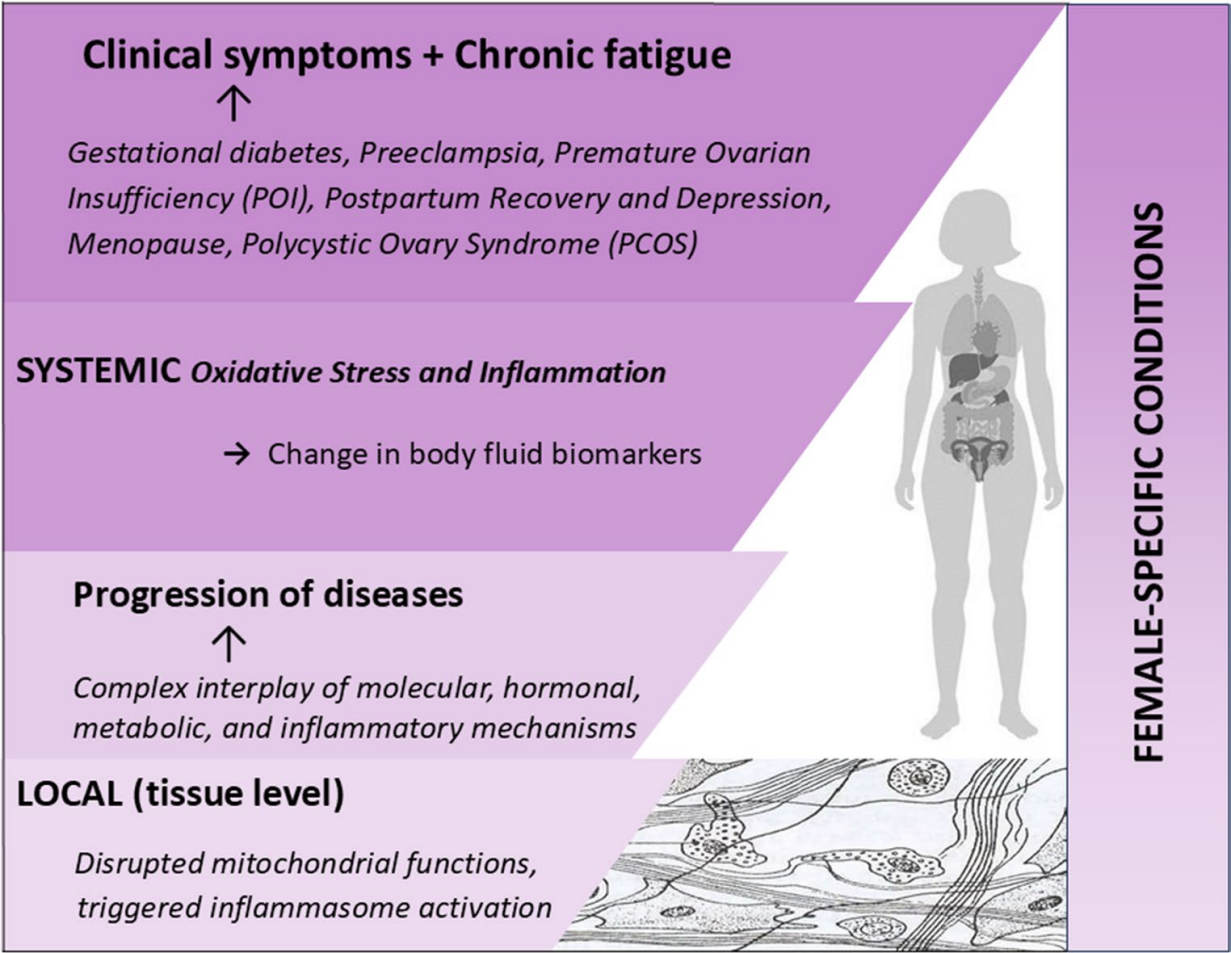
Schematic representation of how impaired mitochondrial function and pro-inflammatory factors contribute to the development and progression of diseases such as preeclampsia and gestational diabetes. The diagram highlights the transition from localised tissue damage to systemic metabolic and inflammatory dysregulation

As these diseases progress, oxidative damage and inflammatory signalling expand beyond the local tissue level to the systemic level, affecting the entire organism as reflected in the issue-dedicated Fig. 2. 3PM innovation is considered essential to protect affected individuals against disease progression. In order to be effective, corresponding mitigation measures and rehabilitation programmes should be essentially tailored to individualised patient profiles (IPP) [6, 7, 10, 21, 22]. Due to high complexity of IPP, an application of AI is of the paramount importance.

## High demand conditions impairing energy metabolism

Fluctuating hormone levels and low-grade chronic inflammation represent major health challenges throughout a woman’s life. These physiological stressors can disrupt cellular homeostasis and increase metabolic demands.

### Hormonal status

In steroidogenic tissues such as ovaries and the adrenal cortex, mitochondria play an essential role in the generation of steroid hormones including the female sex hormones. The synthesis of estrogen and progesterone starts from cholesterol in the mitochondria and is completed in the endoplasmic reticulum during the process of steroidogenesis. These hormones are, in turn, able to modulate mitochondrial activities on the systemic level [11].

During puberty, rising levels of female sex hormones, primarily estrogen and progesterone, play a significant role in modulating mitochondrial function across various tissues. Estrogen binds to specific intracellular receptors leading to changes in expression of both nuclear and mitochondrial genes, especially via the ERβ receptor [23]. Estrogen stimulates the production of important transcription factors such as NRF-1 and PGC-1α, which promote the expression of genes involved in mitochondrial biogenesis, dynamics, and mitochondrial metabolism enhancing mitochondrial efficiency [24–28]. Progesterone acts via mitochondria-specific receptor (PR-M). Progesterone enhances mitochondrial membrane potential and respiratory efficiency [29].

The hormonal effects of estrogen and progesterone help regulate not only energy metabolism but also the production of reactive oxygen species and the ability to cope with their impacts [11, 24]. The overall effect of these hormonal changes is a systematic enhancement of mitochondrial function, supporting the increased energy demands and developmental processes characteristic of female puberty [24, 25, 28, 30] This hormonal regulation of mitochondria is thought to underlie some of the observed sex differences in disease susceptibility and ageing, as the protective effects of estrogen on mitochondria diminish after menopause [11, 27].

Women’s hormones fluctuate throughout the menstrual cycle and significantly modulate mitochondrial function in different tissues. During the follicular phase, when estrogen levels are high, estrogen enhances mitochondrial respiration, particularly in neural synapses and other tissues, leading to increased energy production and improved cellular function [24, 31–33]. This effect is less pronounced during the luteal phase, when progesterone is higher and estrogen is lower [31].

The interplay between estrogen and progesterone, further modulates these mitochondrial effects, although the precise mechanisms are still being studied. Both hormones can enhance mitochondrial efficiency and energy production, but their effects can differ by tissue and context; estrogen generally boosts mitochondrial respiration and biogenesis, while progesterone’s effects can be either supportive or inhibitory depending on the tissue and hormonal balance [25, 34–36].

These cyclical hormonal changes may also impact women’s vulnerability to stress, inflammation, and age-related diseases, as mitochondrial efficiency and resilience are closely tied to hormone levels [25, 35]. Thus, the cyclical changes in women’s hormones orchestrate mitochondrial activity, supporting reproductive health and broader cellular functions. During periods of low estrogen, such as menopause, mitochondrial function does decline, which increases vulnerability to age-related diseases [34, 37, 38].

### Low-grade chronic inflammation

During inflammatory processes, changes occur in mitochondrial bioenergetics, accompanied by increased production of reactive oxygen species (ROS), which in turn further modulate the course of inflammation. Pro-inflammatory cytokines such as TNF-α, IL-1β, and IFN-γ do affect mitochondrial function at several levels. TNF-α, IL-1β, IL-17, IL-18, and IFN-γ activate membrane receptors and downstream pathways (such as NF-κB, STAT1, and MAPKs), which can lead to mitochondrial dysfunction, disrupting energy production, increased calcium influx, and further ROS generation. This overproduction of ROS contributes to oxidative stress, particularly in inflammatory conditions [39–41]. At controlled, low levels, ROS are essential for normal cellular signalling and physiological processes, helping to maintain homeostasis and regulate gene expression [42, 43]. However, increased production of ROS exerts a dual effect, on one hand, excessive ROS can cause oxidative damage to DNA, lipids, and proteins, thereby exacerbating cellular stress and amplifying the inflammatory response on the other hand, ROS act as messengers in intracellular signalling pathways, contributing to the activation of transcription factors such as NF-κB or HIF-1α and stimulate inflammation again [44, 45].

Pro-inflammatory cytokines IFN-γ and IL-1β increase ROS through NADPH oxidase. IFN-γ directly activates NADPH oxidase, while IL-1β is more indirectly associated with NADPH oxidase activity and ROS production [46–48]. TNF-α induces ROS production in the human body through several interconnected cellular mechanisms. In some cell types, TNF-α stimulates NADPH oxidase, resulting in the production of superoxide anions [49]. In one pathway, TNF-α activates adaptor proteins such as RIP1, TRAF2, and caspase-8. Caspase-8 can cleave Bid, which interacts with mitochondrial proteins such as Bcl-xL and Bax, leading to mitochondrial outer membrane permeabilisation, reduced membrane potential, and cytochrome c release. This mitochondrial dysfunction often increases ROS production, which amplifies apoptotic signalling [50–53].

IL-1β activates also the inducible isoform of nitric oxide synthase (iNOS), resulting in elevated nitric oxide (NO) levels. Excess of NO can react with ROS to form peroxynitrite, a highly reactive molecule that damages mitochondrial components and disrupts their function [40, 54]. Impact on mitochondrial function by increased ROS and NO from NADPH oxidase and iNOS activation lead to loss of mitochondrial membrane potential, reduced ATP production, and activation of cell death pathways (e.g., caspase activation) [54, 55]. The combination of cytokines can amplify ROS and NO production, further impairing mitochondrial function, contributing to inflammatory conditions and even promoting cell death [39, 40, 54].

## Hormonal transition phases throughout women’s lives

Several distinct transition phases (puberty, pregnancy-related cardiovascular stress, lactation, and menopause) are characterised by profound shifts in hormonal production that drive major changes in phenotype, physiology, and metabolic regulation, representing a unique physiological challenge with specific implications for mitochondrial function, inflammatory pathways, and long-term health outcomes.

### Puberty

In girls, the intense hormonal changes and rapid growth of puberty place substantial demands on bones, muscles, the cardiovascular system, and metabolic pathways. While these changes are essential for healthy development, they can also temporarily stress tissues and systems, especially if compounded by poor nutrition, intense physical activity, or underlying health issues. Estrogen and growth hormone (GH) interact closely to drive the pubertal growth spurt and changes in body composition unique to females. These hormones stimulate protein synthesis, muscle mass increase, bone mineralisation, and changes in body composition, requiring tissues to adapt quickly to new growth and metabolic demands [56–61].

Several pathophysiological conditions and diseases that can emerge or worsen during puberty are linked to impaired mitochondrial functions, increased mitochondrial reactive oxygen species (ROS) production, and the development of chronic inflammation. Metabolic disorders such as obesity and diabetes, which are increasingly seen in adolescents, are associated with mitochondrial dysfunction, chronic low-grade inflammation, and excessive ROS generation [62–64].

Autoimmune diseases, including type 1 diabetes and juvenile forms of conditions like systemic lupus erythematosus, also involve mitochondrial dysfunction and the release of mitochondrial components that trigger inflammatory responses, further increasing ROS and perpetuating inflammation [65]. Cardiovascular risk factors that may begin in adolescence, such as hypertension and hyperlipidemia, are similarly linked to oxidative stress and mitochondrial abnormalities, contributing to vascular inflammation and early atherosclerosis [62, 63, 66]. In these conditions, excessive ROS from dysfunctional mitochondria not only damages mitochondrial proteins and DNA but also acts as a signal to amplify inflammatory pathways, a process sometimes referred to as “mito-inflammation”. This chronic inflammatory, creating a cycle that underlies the progression of many chronic diseases with roots in adolescence [63, 66–68].

### Cardiovascular stress during pregnancy

Cardiovascular stress during pregnancy refers to the significant physiological and psychological demands placed on the maternal cardiovascular system. Pregnancy acts as a natural “stress process” revealing both the body’s adaptive mechanisms and potential Insufficiency. In the time of pregnancy can thus reveal underlying subclinical cardiovascular and metabolic abnormalities that can then increase the risk of pregnancy disorders like preeclampsia and later-life cardiovascular disease. Failure to adapt to these changes can result in complications for both mother and child, with implications for long-term health. Cardiovascular stress is reflected in the level of physiological changes and on psychological level and that is how from short-term so long-term point of view. Physiological changes like hemodynamic adaptations induces increased cardiac output, arterial compliance, and vasodilation, with a marked decrease in systemic vascular resistance and blood pressure. These changes support fetal growth and ensure adequate uteroplacental circulation. By postpartum, most cardiovascular parameters return to pre-pregnancy levels. Inadequate adaptation can lead to disorders such as preeclampsia, gestational hypertension, and heart failure, which are associated with increased maternal and fetal morbidity [69–71].

Psychological stress and anxiety during pregnancy are linked to adverse cardiovascular outcomes. These factors can exacerbate physiological stress, increasing the risk of complications [72].

The postpartum period is crucial for lifestyle interventions and early diagnosis of chronic conditions. However, many women do not receive adequate follow-up or counseling regarding their increased long-term CVD risk [73, 74].

### Lactation

Lactation under high metabolic demand, such as breastfeeding multiples (twins), with insufficient postpartum recovery, simultaneous pregnancy, breastfeeding during infection or increased physical activity, poses extra physical and emotional stress for breastfeeding mothers, making breastfeeding more difficult and increasing the risk of early cessation [75–79].

To meet metabolic demands breastfeeding multiples, the maternal body undergoes substantial physiological adaptations, including increased mobilisation of fat and muscle stores, enhanced intestinal absorption of nutrients, and even resorption of maternal bone to supply enough calcium for milk production [80–82]. These heightened metabolic requirements can challenge the mother’s ability to maintain her own health, especially if her dietary intake is insufficient or if she starts lactation with depleted nutrient stores If the mother cannot adequately adapt, there is a higher risk of metabolic imbalances, such as ketosis, and potential long-term impacts on her metabolic and bone health [80, 81]. To meet the high energy demands, mitochondria in key tissues like the liver and skeletal muscle increase their respiratory activity and efficiency, upregulate biogenesis, and adjust antioxidant enzyme activity. These adaptations generally support enhanced ATP production but can also lead to increased production of reactive oxygen species (ROS), especially if antioxidant defences are insufficient, raising the risk of oxidative damage and chronic inflammation [83–87]. However, successful lactation is also associated with long-term improvements in maternal metabolism and mitochondrial function, supporting the “lactation reset hypothesis”, which suggests that these adaptations can confer lasting metabolic benefits if the process is not excessively strained [88]. On the other hand, malnutrition during lactation can significantly impair mitochondrial function. This dysfunction manifests as reduced mitochondrial energy production, disrupted electron transport chain activity, and increased production of reactive oxygen species (ROS), which further activates inflammatory pathways, including the inflammasome, and can drive chronic inflammation [89].

In animal studies, malnutrition during lactation has been shown to disturb mitochondrial gene expression and steroid hormone production in ovarian cells, impairing reproductive function, while also causing long-lasting changes in pancreatic islet mitochondria that may predispose offspring to metabolic diseases like diabetes [89–91].

These findings underscore that mitochondrial dysfunction is a central link between malnutrition and the negative health effects observed during and after lactation, affecting both maternal and offspring well-being.

### Menopause

Menopause is defined by the permanent cessation of menstruation due to the loss of ovarian follicular activity, typically occurring between ages 46 and 55. The most significant physiological change is a marked decline in estrogen and progesterone production, resulting from the depletion of ovarian follicles. This hormonal shift leads to increased levels of follicle-stimulating hormone (FSH) and decreased levels of inhibin B and anti-Müllerian hormone (AMH) [92]. These changes cause a range of symptoms and physiological effects, increased fat mass, decreased lean mass, and a redistribution of body fat, sleep disturbances, and others [93, 94]. Estrogen important hormonal regulators of inflammation partly by signaling through its receptors (ER-α and ER-β) to suppress inflammatory pathways and maintain mitochondrial efficiency. Loss of estrogen disrupts these protective mechanisms, leading to heightened activation of the innate immune system, increased inflammasome activity, and systemic inflammation, which can affect both peripheral tissues and the central nervous system [95]. Additionally, postmenopausal women experience increases in adiposity and adipokines like leptin and adiponectin, which further promote low-grade chronic inflammation and metabolic disturbances [96]. The combined effects of increased inflammation, oxidative stress from elevated ROS, and mitochondrial dysfunction create a cellular environment that raises the risk for chronic diseases such as cardiovascular disease and neurodegenerative disorders [96–98].

## Reciprocity between mitochondrial health status and female gender-specific disorders – prominent examples

**Fig. 3:**
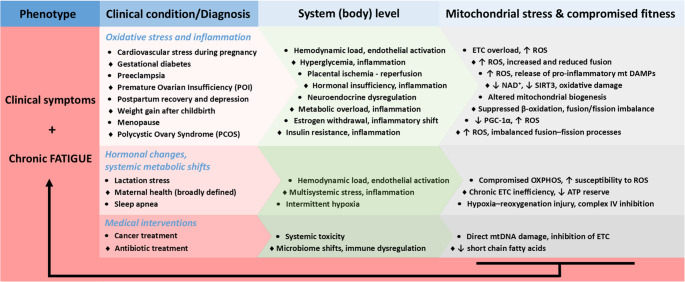
System – mitochondria interplay underlying chronic fatigue in female-specific clinical contexts. This scheme links frequent female-relevant clinical conditions with systemic (whole-body) stressors and their convergent impact on mitochondrial function. Abbreviations: ATP – adenosine triphosphate; ETC – electron transport chain; mtDNA – mitochondrial DNA; NAD⁺ – nicotinamide adenine dinucleotide; OXPHOS – oxidative phosphorylation; PCOS – polycystic ovary syndrome; PGC-1α – peroxisome proliferator-activated receptor gamma coactivator-1 alpha; POI – premature ovarian insufficiency; ROS – reactive oxygen species; SIRT3 – sirtuin 3 (mitochondrial deacetylase); DAMPs – damage-associated molecular patterns

Diverse hormonal transitions, metabolic challenges, inflammatory states, and medical interventions collectively increase mitochondrial stress, reduce bioenergetic fitness, and promote oxidative damage. Figure 3 illustrates how cumulative mitochondrial dysfunction provides a unifying mechanistic basis for the development and persistence of chronic fatigue across different phenotypes and life stages, highlighting mitochondria as central integrators of systemic health and disease vulnerability. The persistent energetic mismatch manifests clinically as chronic fatigue, thereby linking diverse phenotypes at the systemic level to a common mitochondrial mechanism highlighted in Fig. 3.

### Gestational diabetes

Gestational diabetes mellitus (GDM) typically develops in pregnancy due to a combination of increased insulin resistance and insufficient pancreatic β-cell compensation, with risk factors including obesity, advanced maternal age, and family history of diabetes. GDM affects up to ∼1 in 6 pregnancies worldwide [99, 100]. GDM is characterised by elevated blood glucose and fatty acid levels, which disrupt normal cellular metabolism. These metabolic changes induce mitochondrial dysfunction in key tissues such as the placenta and adipose tissue, leading to reduced ATP production and impaired mitochondrial dynamics, including decreased expression of mitochondrial fusion proteins [99, 101, 102]. Mitochondrial dysfunction in GDM is closely linked to increased production of reactive oxygen species (ROS), particularly in placental macrophages, which contributes to oxidative stress and cellular damage [100, 103]. The excess of ROS further activates inflammatory pathways, creating a vicious cycle where inflammation and oxidative stress reinforce each other, often mediated by the NF-κB signaling pathway [104]. These processes not only impair maternal insulin sensitivity but also increase the risk of adverse pregnancy outcomes and long-term metabolic complications for both mother and child [99, 100, 105].

Obesity in pregnancy elevates fatty acid levels in the placenta, damaging mitochondrial membranes, increasing ROS, and promoting a cycle of mitochondrial dysfunction and chronic inflammation that can affect both mother and fetus. Chronic inflammation, often measured by markers like C-reactive protein, is both a cause and consequence of this oxidative stress, and is exacerbated by poor sleep and metabolic disturbances [106, 107].

### Preeclampsia

Preeclampsia is set under conditions of abnormal placentation, particularly inadequate trophoblast invasion and poor remodelling of the maternal spiral arteries, which leads to placental ischemia, hypoxia and intravascular inflammation [108]. This hypoxic environment impairs mitochondrial function in placental cells, resulting in reduced energy production and increased generation of reactive oxygen species (ROS) [108, 109]. Elevated ROS levels cause oxidative stress, which stabilises hypoxia-inducible factors and promotes the release of antiangiogenic factors, further contributing to endothelial dysfunction and systemic inflammation [110–112]. Mitochondrial dysfunction in preeclampsia is marked by abnormal mitochondrial morphology, impaired oxidative phosphorylation, and decreased activity of key electron transport chain proteins, especially in early-onset cases [109, 113]. Oxidatively damaged mtDNA released from stressed trophoblasts can activate innate sensors (e.g., TLR9, NLRP3 inflammasome), amplify inflammatory signalling and contribute to endothelial dysfunction — central features of preeclampsia pathophysiology [114].

### Cardiovascular stress during pregnancy with complications

Cardiovascular stress during pregnancy can be triggered by conditions such as gestational hypoxia, obesity, poor sleep, psychological stress, and metabolic disorders like gestational diabetes and preeclampsia. These stressors disrupt the maternal cardiovascular system and place increased demands on mitochondrial function, particularly in the placenta and fetal heart [115–120]. Hypoxic conditions, impair mitochondrial respiration and increase reactive oxygen species (ROS) production, especially in male fetuses, leading to oxidative stress that is not always mitigated by antioxidant treatments [121]. Thus, cardiovascular stress during pregnancy, through its effects on mitochondrial health and ROS production, plays an important role in the development of chronic inflammation and adverse pregnancy outcomes.

### Polycystic ovary syndrome (PCOS)

Polycystic Ovary Syndrome (PCOS) is a polygenic and multifactorial, heterogeneous disorder affecting 5–20% of women of reproductive age and is characterised by hyperandrogenism, ovulatory dysfunction, and polycystic ovarian morphology [122, 123]. The pathophysiology involves a combination of genetic and environmental factors, with central roles for hormonal imbalances - particularly increased luteinising hormone (LH) secretion due to altered gonadotropin-releasing hormone (GnRH) pulsatility - and insulin resistance, which leads to compensatory hyperinsulinemia, all of which contribute to its development and progression [122–124]. Chronic low-grade inflammation and adiposity-related mechanisms further exacerbate the syndrome, with increased production of inflammatory cytokines and altered adipose tissue function [123, 125]. Because PCOS is associated with metabolic complications such as obesity, dyslipidemia, impaired glucose tolerance, and an increased risk of type 2 diabetes and cardiovascular disease [122, 123]. This in turn negatively impact mitochondrial health and function. Mitochondrial dysfunction in PCOS is characterised by mutations in mitochondrial DNA, impaired oxidative phosphorylation, reduced ATP production, and abnormal mitochondrial dynamics, especially in ovarian granulosa cells [126–130]. This dysfunction results in increased production of reactive oxygen species (ROS), which further damages mitochondrial components and cellular structures, perpetuating oxidative stress [131–133]. The excess ROS and mitochondrial damage activate inflammatory pathways, leading to persistently elevated levels of inflammatory markers such as TNF-α, CRP, and various interleukins, establishing a state of chronic inflammation [127, 132]. Thus, PCOS is both a cause and consequence of mitochondrial dysfunction, increased ROS production, and chronic inflammation, with these processes tightly interlinked in its pathophysiology.

## Side effects of medical interventions in relation to mitochondrial health

### Antibiotic treatment (microbiome disruption)

Regular antibiotic treatment can cause significant disruption to the gut microbiome, leading to a marked reduction in microbial diversity and the depletion of beneficial bacteria such as *Bifidobacterium* and *Lactobacillus* [134–137]. The extent of disruption depends on factors such as the type, duration, and spectrum of antibiotics used, as well as individual host factors like age, diet, and underlying health conditions [135, 137, 138]. While some aspects of the microbiome may recover within weeks to months after treatment, certain species can remain undetectable or at reduced levels for extended periods, and the overall community structure may not fully return to its original state even after six months [134, 137].

The human gut microbiome produces several key metabolites that are indispensable for mitochondrial metabolic processes. Among the most important are short-chain fatty acids (SCFAs) such as acetate, propionate, and butyrate, which are generated from the fermentation of dietary fibers and serve as major energy sources for colonocytes and can influence mitochondrial energy metabolism throughout the body [139–142]. Secondary bile acids, produced by microbial transformation of primary bile acids, play roles in regulating mitochondrial function and lipid metabolism [139, 143]. Tryptophan and its microbial-derived indole metabolites are involved in modulating mitochondrial oxidative stress and energy production [139–141].

### Cancer treatment

Among women worldwide, the most frequently diagnosed malignancies include breast, colorectal, cervical, ovarian, endometrial, and thyroid cancers. Their incidence rises markedly beginning in the fourth and fifth decades of life, coinciding with major hormonal transitions, such as perimenopause and menopause, that alter estrogen and progesterone signalling and modulate metabolic and mitochondrial functions (Bray et al., 2024; Sung et al., 2021). These endocrine changes affect oxidative stress, immune responses, and cellular energy metabolism, thereby contributing to both the initiation and progression of hormone-related tumors, particularly breast and endometrial cancer (Labrie et al., 2017; Simpson & Brown, 2022).

Multiple lines of evidence indicate that standard and advanced cancer treatments can impair mitochondrial health in non-cancer cells. Chemotherapy and radiotherapy have been shown to increase mitochondrial reactive oxygen species (mtROS), damage mitochondrial DNA (mtDNA) and telomeric DNA, reduce mitochondrial membrane potential and alter mitochondrial dynamics [144]. In particular, chemotherapy-associated fatigue and skeletal muscle weakness (common in survivors) have been linked to mitochondrial dysfunction in muscle [145]. Impaired mitochondrial biogenesis, abnormal mitochondrial dynamics (fusion/fission imbalance), and defective mitophagy (mitochondrial quality control) may predispose to persistent mitochondrial dysfunction, contributing to fatigue, cardiotoxicity, neuropathy, metabolic dysregulation and premature ageing in cancer survivors [10].

## Recommended tools for the health status monitoring followed by evidence-based individualised treatment and protective measures

Based on the above presented scientific evidence, it can be concluded that physiological hormonal changes specifically during female life create conditions with high demands on mitochondrial plasticity. When physiological limits and compensatory capacity are exceeded, mitochondrial impairment may develop as a result of an excessive oxidative stress and pro-inflammatory activation. The major risks of these processes are the compromised mitochondrial functionality and chronification of both – inflammation and pain which are abundantly described e.g. for the Flammer Syndrome Phenotype (FSP) carriers [22, 146]. These crucial aspects are further discussed by the below provided patient case analyses (subchapter 5.3 “Phenotyping is of great utility for health risk assessment in vulnerable subpopulation: Preliminary outcomes of the clinical study applied Flammer syndrome phenotyping to the pre-menopausal breast cancer patient cohort”).

From a diagnostic standpoint, it is crucial to recognise conditions in which compensatory mechanisms fail to sustain homeostatic balance, and the levels of key metabolites, enzymes, and signalling molecules deviate from the physiological range. Monitoring robust biochemical marker panels combined with systemic diagnostic tools, such as cell-free mtDNA patterns, provides an opportunity [5–8, 10, 146–149].

1. to shift the paradigm from reactive to proactive medical care saving lives and resources.
2. to introduce innovative screening programmes focused on suboptimal health conditions with reversible damage of affected individuals in primary care.
3. to offer health risk assessment in primary and secondary care followed by treatment algorithms tailored to individualised patient profile.
4. to protect individuals against health-to-disease transition in primary care and against disease progression in secondary care.
5. to monitor health status stability of patients.
6. to stratify patients for targeted therapeutic intervention.
7. to monitor efficacy of the applied therapeutic approach.
8. to create individualised pre- and rehabilitation programmes.
9. for digital health monitoring.
10. to promote evidence-based anti-ageing and rejuvenation programmes with significantly improved individual outcomes.
11. to increase quality of physical fitness coaching and exercise intervention as recommended by the field-recognised experts.

### Biochemical tests

Routine biochemical assays relevant to tissue injury caused by oxidative stress secondary to mitochondrial dysfunction typically include markers of oxidative damage to biomolecules, such as lipid peroxidation products (malondialdehyde [MDA], 4-hydroxynonenal [4-HNE]), protein oxidation and nitration products (carbonylated proteins, 3-nitrotyrosine), and indices of nucleic acid oxidation (8-hydroxy-2′-deoxyguanosine [8-OHdG]). Complementary assessment of endogenous antioxidant capacity, including reduced and oxidised glutathione (GSH/GSSG ratio), superoxide dismutase (SOD), catalase, and glutathione peroxidase activities, provides additional diagnostic information on the balance between pro-oxidant burden and compensatory defence mechanisms.

When the values of the above-mentioned parameters deviate from the physiological range, accompanied by elevations of markers indicative of cellular leakage and mitochondrial impairment (e.g., lactate dehydrogenase [LDH], creatine kinase [CK], aspartate aminotransferase [AST], alanine aminotransferase [ALT]), this indicates a failure of compensatory mechanisms, which becomes manifested by clinically detectable symptoms.

In gestational diabetes mellitus (GDM), elevated MDA levels (lipid peroxidation) have been reported, accompanied by reduced GSH and SOD activity [150]. Furthermore, higher levels of protein oxidative markers (protein carbonyls [PCO], advanced oxidative protein products [AOPPs]) and lipid peroxidation products (8-iso-PGF2α), along with decreased antioxidant enzymes such as GPX-3 and PON1 were demonstrated [151, 152].

The pathogenesis of preeclampsia is closely linked to severe tissue damage and oxidative stress. LDH is frequently elevated in women with preeclampsia, particularly in severe cases, and one study identified LDH and ALT as markers associated with disease severity (PubMed). Liver function tests, including AST, ALT, bilirubin, and LDH, are significantly altered in preeclampsia compared with normal pregnancies (PubMed). Moreover, higher AST and ALT levels measured early in pregnancy (before week 20) have been associated with the later development of severe preeclampsia, although their diagnostic sensitivity and specificity remain limited (PubMed).

In menopause, the physiological loss of estrogens is associated with increased oxidative stress, as evidenced by elevated lipid peroxidation markers (e.g., MDA), reduced antioxidant capacity (lower GSH, SOD, CAT, GPx). Some markers (like protein carbonyls) are less well studied in this context, and data for nucleic acid oxidation indices (e.g. 8-OHdG) are currently limited. Postmenopausal women have significantly higher serum and salivary MDA vs. premenopausal women [153]. MDA levels correlate positively with age and duration since menopause [154]. Studies show a decrease in reduced glutathione (GSH) and lower activities of SOD, catalase, and glutathione peroxidase (GPx) in perimenopausal and postmenopausal women compared to reproductive-age women [155]. Hormone Replacement Therapy (HRT or estrogen+progestin) tends to mitigate some of the decline: women on HRT show higher SOD activities and total antioxidant power than those without [156].

Evidence from animal and human studies indicates that during lactation under high metabolic demand or malnutrition, markers of oxidative stress such as MDA and 4-HNE increase in certain tissues, while antioxidant defenses (e.g. GSH, SOD, GPx, CAT) tend to decrease. In severe energy deficit or malnutrition, there is also elevated DNA oxidation (e.g. 8-OHdG) and protein oxidation.

Puberty is a period of rapid hormonal change, growth, and metabolic reprogramming, which can increase oxidative stress; however, in young, healthy individuals these stressors are generally well compensated by robust antioxidant defenses. Evidence suggests that during this transitional period, markers of DNA oxidation and pro-oxidative metabolites may transiently increase, while antioxidant systems (such as SOD, GPx, catalase, and total antioxidant capacity) adapt and maintain redox balance. A case-control study in girls with precocious puberty found alterations in catalase and SOD activity and total antioxidant/oxidant status compared to healthy peers, consistent with the concept of compensatory upregulation of defenses in response to hormonal changes [157].

Antibiotic treatment causes acute disruption of the gut microbiome, which perturbs microbial metabolism (e.g. reducing SCFA production), increases luminal oxygen and electron acceptors, and shifts redox potential, thereby generating oxidative stress in the gut environment [158]. While robust antioxidant defenses and epithelial barrier functions may initially compensate, prolonged or repeated antibiotic exposure can overwhelm these mechanisms, potentially leading to increased biomolecular damage (lipid peroxidation, protein and DNA oxidation) and impaired recovery. Direct human data on systemic oxidative damage markers (e.g. MDA, 8-OHdG, ALT/AST) is still limited [159]. It is evident that restoration of the gut microbiome is crucial in this context.

### Systemic diagnostics utilising conceptual and technological innovation by health condition-specific cell-free MtDNA patterns

Mitochondrial DNA can be assessed as an intracellular parameter, reflecting the copy number of mitochondrial DNA within the cell (mtDNA-CN), which serves as an indicator of mitochondrial abundance and functional capacity. However, a reliable indicator of mitochondrial health and homeostasis is an extracellular parameter, referring to free mitochondrial DNA or its fragments detectable in blood plasma, commonly termed circulating cell-free mitochondrial DNA (ccf-mtDNA). Ccf-mtDNA reflects systemic mitophagy levels originating from tissue damage, cellular stress, and inflammatory activation [149]. High ccf-mtDNA levels may indicate active compensatory regulation of mitochondrial homeostasis under stress conditions, while low levels may show mitochondrial burn-out [7]. Cell-free mtDNA is detectable also in tear fluid offering non-invasive alternative to blood for diagnostics of mitochondrial health [5, 7] with the advantage of greater stability in molecular patterns compared to blood allowing long-term monitoring of to track changes in mitochondrial health over time. Mitochondrial damage and impairments can be detected early at the stage of reversible damage to health, which is crucial for tailoring preventive measures and individualised treatments [160–162].

In gestational diabetes mellitus (GDM), higher plasma free mtDNA levels were noted in GDM patients compared to control group of normal pregnant women, which may reflect increased mitochondrial damage and release into circulation [163]. Elevated levels of ccf-mtDNA have been detected in GDM patients, particularly in the first trimester. This elevation is associated with heightened oxidative stress, leading to mitochondrial damage and the release of ccf-mtDNA into the bloodstream. The presence of ccf-mtDNA can activate immune responses, including the NLRP3 inflammasome, contributing to systemic inflammation characteristic of GDM [164, 165].

Multiple studies report that placental mtDNA copy number (mtDNA-CN) and mitochondrial mass are altered in preeclampsia, with several papers showing increased mtDNA-CN in early-onset/severe preeclampsia placentas, whereas other cohorts (or analyses of maternal blood) report decreased or variable mtDNA-CN. This heterogeneity likely reflects differences in preeclampsia subtype (early vs. late onset), tissue analysed (placenta vs. maternal whole blood or plasma), gestational timing and laboratory methods [166, 167].

Cohort analyses indicate that maternal circulating mtDNA dynamics are abnormal in preeclampsia. Some studies report reduced plasma ccf-mtDNA concentrations (and altered clearance) in preeclampsia compared with gestational-age matched controls [168], while others find elevated maternal serum mtDNA [169].

These findings suggest altered mtDNA trafficking (release from damaged trophoblast, packaging in extracellular vesicles, or impaired clearance) in preeclampsia, which is highly indicative for innovative diagnostics.

Finally, the reciprocity between the quality of mitochondrial health and homeostasis on one hand and physical fitness and exercise intervention on the other hand has been proposed as being instrumental for individualised health monitoring within pre- and rehabilitation programmes in secondary care. The proposed conceptual and technological innovation utilises systemic effects, tear fluid multi-omics, mitochondria as a vital biosensor and AI-based multimodal data interpretation [149].

### Phenotyping is of great utility for health risk assessment in vulnerable subpopulation: Preliminary outcomes of the clinical study applied Flammer syndrome phenotyping to the pre-menopausal breast cancer patient cohort

Per scientific evidence, breast cancer (BC) is considered a pathology which is more prevalent in post-menopausal women [170]. However, BC incidence in young female subpopulations is steadily increasing. This trend became particularly remarkable in the 21 st century presenting a socio-economic disaster and demanding a fundamental revision of the currently applied healthcare concepts to reverse the trend. An “across-the-board” population screening is not doable from the economic point of view. In contrast, patient phenotyping to low-costs performed by primary healthcare givers is a promising proactive approach to identify vulnerable individuals for protecting them against health-to-disease transition [4].

Flammer syndrome phenotyping has been proposed for a spectrum of pathologies which the FSP carriers are predisposed to, due to strongly pronounced phenotype-specific psychosomatic patterns (sympathetic overdrive), persistently disturbed microcirculation, frequently reduced sleep quality and duration, chronification of the low-grade inflammation and compromised mitochondrial health, among others [146]. The FSP onset is associated with the puberty and can be detected early in life that is instrumental for cost-effective proactive 3PM healthcare approach.

The nominated EPMA expert group carries out a study focused on the pre-menopausal FSP carriers diagnosed with BC. Preliminary results demonstrate high co-incidence of the FSP in this patient cohort. Moreover, individual outcomes indicate characteristic for the pre-menopausal BC patients’ correlations: the more of below listed risk factors are pronounced and simultaneously appear in their dossier, the younger is the patient diagnosed with BC, namely.

- high FSP score.
- compromised lifestyle.
- low physical activity.
- psycho-somatic complications (anxiety, depression, panic attacks, etc.)
- chronic fatigue.
- chronicity of inflammation.
- slowly healing wounds.
- earlier diagnosed benignant tissue-transformations.
- cancer-diagnosed grand/parents (not necessarily breast and/or prostate cancer).

demonstrating synergies between genetic predisposition and phenotype-relevant health risks on one hand, and on the other hand, per evidence, reduced mitochondrial functionality and shifted mitochondrial homeostasis as overviewed elsewhere [161, 171]. Below we exemplify patient cases to illustrate these conclusions.

#### Patient case 1 analysis

55 years old pre-menopausal women diagnosed with BC; following health risk factors are recorded:

- highly scored FSP (including arrhythmias, migraine attacks without aura, disturbed microcirculation, reduced sleep duration, reduced thirst perception, increased pain perception, eye and vaginal dryness).
- chronic fatigue.

#### Patient case 2 analysis

48 years old pre-menopausal women diagnosed with BC; following health risk factors are recorded:

- highly scored FSP (low BMI, persistently disturbed microcirculation, reduced sleep quality and duration, increased pain perception, migraine attacks with aura, tinnitus, increased sensitivity to medication, pronounced eye and vaginal dryness).
- decreased physical activity.
- psycho-somatic complications (anxiety, depression, panic attacks, etc.)
- chronic fatigue.
- chronicity of inflammation.
- slowly healing wounds.
- grandfather diagnosed with bladder cancer.

#### Patient case 3 analysis

44 years old pre-menopausal women diagnosed with BC; following health risk factors are recorded:

- highly scored FSP (low BMI, persistently disturbed microcirculation, dizziness, insomnia, increased pain perception, migraine attacks with aura, pronounced vaginal dryness).
- compromised lifestyle.
- low physical activity.
- chronic fatigue.
- pronounced chronic systemic inflammation.
- slowly healing wounds.

#### Patient case 4 analysis

40 years old pre-menopausal women diagnosed with BC; following health risk factors are recorded:

- highly scored FSP (low BMI, frequent arrhythmias, migraine attacks with aura, disturbed microcirculation, poor sleep quality, reduced thirst perception, increased pain perception, pronounced eye and vaginal dryness).
- PCOS.
- low physical activity.
- chronic fatigue.
- pronounced anxiety and depression.
- pronounced chronic systemic inflammation.
- slowly healing wounds.
- grandmother diagnosed with BC; father diagnosed with lung cancer.

## Therapeutic and preventive strategies targeting mitochondrial health

Maintaining optimal mitochondrial function is increasingly recognised as an important point of disease prevention and therapy across a broad range of pathologies. Therapeutic and preventive strategies aimed at supporting mitochondrial health focus on reducing oxidative stress, improving oxidative phosphorylation efficiency, enhancing mitochondrial biogenesis, and promoting mitophagy to remove damaged mitochondria. Mitochondrial dysfunction, oxidative stress, and chronic low-grade inflammation represent shared mechanistic hallmarks of female-specific metabolic and hormonal disorders, including preeclampsia, gestational diabetes, polycystic ovary syndrome (PCOS), and menopause-associated metabolic decline.

A promising therapeutic and preventive direction lies in the application of mitochondria-relevant nutraceuticals that help restore mitochondrial homeostasis, improve redox balance, and modulate inflammatory signalling. Evidence-based compounds such as coenzyme Q10, L-carnitine, and creatine support mitochondrial energy buffering and oxidative phosphorylation efficiency, while alpha-lipoic acid, vitamins C and E, and N-acetyl-cysteine act as potent antioxidants mitigating ROS-induced damage.

The PPAR–PGC-1α axis has emerged as a central target regulating mitochondrial biogenesis, lipid and glucose metabolism, and redox balance. Synthetic PPAR agonists such as fibrates and thiazolidinediones enhance mitochondrial energy metabolism. Fibrates (PPAR-α agonists) have been shown to increase mitochondrial fatty acid oxidation, enhance mitochondrial biogenesis, and improve ATP production, particularly in heart, liver, and muscle [172]. Thiazolidinediones (PPAR-γ agonists) can promote mitochondrial biogenesis, increase fatty acid oxidation, and improve insulin sensitivity, especially in adipose tissue and muscle [173]. However, their clinical use is limited by adverse systemic effects [174].

In contrast, natural PPAR activators, including resveratrol, quercetin, epigallocatechin gallate (EGCG), and astaxanthin, demonstrate comparable efficacy with better safety features.

Polyphenolic nutraceuticals as resveratrol, quercetin, fisetin, curcumin, kaempferol, and apigenin demonstrate multitargeted actions by activating the AMPK–SIRT1–PGC-1α axis, promoting mitochondrial biogenesis and mitophagy, and attenuating NF-κB-driven inflammatory cascades. Among them, resveratrol and quercetin are strong activators of SIRT1 and PGC-1α signalling, enhancing mitochondrial resilience in metabolic stress conditions, while fisetin promotes effectivity of mitochondrial populations.

## Conclusions and outlook in the framework of 3PM

**Fig. 4:**
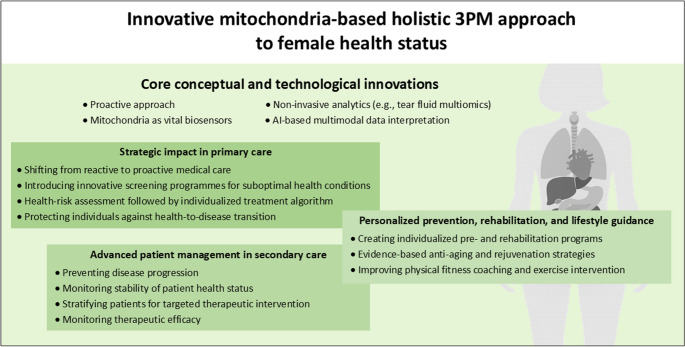
Mitochondria-based holistic 3PM framework for female health. This figure illustrates a mitochondria-centred, predictive, preventive, and personalised medicine (3PM) approach to female health, integrating proactive monitoring, non-invasive bioanalytics, and AI-supported multimodal data interpretation. Utilising mitochondrial biosensorics in a holistic manner, the model links primary and secondary care with personalised prevention, rehabilitation, and lifestyle guidance

The innovative mitochondria-based holistic approach to female health clearly demonstrates how Predictive, Preventive and Personalised Medicine (3PM) can redefine clinical practice. The conceptual innovation is summarised in Fig. 4 highlight that future-oriented healthcare must combine systemic understanding of biological networks with advanced, non-invasive analytics and intelligent data interpretation. Mitochondria emerge as central biosensors that reflect the individual’s health-to-disease trajectory, providing actionable information long before clinically manifested pathology develops. This creates an opportunity to shift from reactive treatment of advanced disease to proactive management of suboptimal health conditions.

In line with the 3PM paradigm, next-generation screening programmes should focus on detecting reversible molecular and functional alterations at preclinical stages. Tools for monitoring mitochondrial biogenesis such as tear-fluid multiomics [175], combined with AI-driven integration of multimodal data, allow clinicians to identify early risk patterns and stratify individuals into tailored intervention pathways. This risk-adapted care model supports personalised decision-making in both primary and secondary care, protecting individuals from disease initiation as well as from progression once pathology is present.

Monitoring health-status stability becomes a cornerstone of long-term management. Regular assessment of mitochondrial performance, metabolic resilience and systemic regulatory mechanisms enables timely therapeutic adjustments and evaluation of treatment efficacy. Such continuous profiling not only guides precision therapies but also supports the development of individualised prehabilitation and rehabilitation programmes. These strategies can be further expanded to evidence-based anti-ageing and rejuvenation interventions with measurable clinical impact.

Looking forward, the integration of mitochondria-centred diagnostics, AI-supported analytics and non-invasive multiomic profiling is expected to advance personalised coaching, lifestyle optimisation and exercise interventions. As a result, 3PM-aligned healthcare will increasingly promote preventive resilience rather than crisis-driven treatment. The outlined innovations therefore represent a crucial step toward sustainable, patient-centred medicine that improves outcomes, preserves quality of life and ensures more efficient use of healthcare resources.

## Data Availability

No datasets were generated or analysed during the current study.

## References

[1] Mauvais-Jarvis F. Sex differences in energy metabolism: natural selection, mechanisms and consequences. Nat Rev Nephrol. 2024;20:56–69. 10.1038/s41581-023-00781-2. 37923858 10.1038/s41581-023-00781-2

[2] Henderson VW, Brinton RD. Menopause and mitochondria: windows into estrogen effects on Alzheimer’s disease risk and therapy. Prog Brain Res. 2010;182:77–96. 10.1016/S0079-6123(10)82003-5. 20541661 10.1016/S0079-6123(10)82003-5 PMC5776041

[3] Sanchez BN, Volek JS, Kraemer WJ, Saenz C, Maresh CM. Sex differences in energy metabolism: a female-oriented discussion. Sports Med. 2024;54:2033–57. 10.1007/s40279-024-02063-8. 38888855 10.1007/s40279-024-02063-8

[4] Wang W, Yan Y, Guo Z, Hou H, Garcia M, Tan X, et al. All around suboptimal health – a joint position paper of the Suboptimal Health Study Consortium and European Association for Predictive, Preventive and Personalised Medicine. EPMA J. 2021;12:403–33. 10.1007/s13167-021-00253-2. 34539937 10.1007/s13167-021-00253-2 PMC8435766

[5] Golubnitschaja O, Kapinova A, Sargheini N, Bojkova B, Kapalla M, Heinrich L, et al. Mini-encyclopedia of mitochondria-relevant nutraceuticals protecting health in primary and secondary care—clinically relevant 3PM innovation. EPMA J. 2024;15:163–205. 10.1007/s13167-024-00358-4. 38841620 10.1007/s13167-024-00358-4 PMC11148002

[6] Shao Q, Ndzie Noah ML, Golubnitschaja O, Zhan X. Mitochondrial medicine: from bench to bedside 3PM-guided concept. EPMA J. 2025;16:239–64. 10.1007/s13167-025-00409-4. 40438494 10.1007/s13167-025-00409-4 PMC12106218

[7] Golubnitschaja O, Polivka J, Potuznik P, Pesta M, Stetkarova I, Mazurakova A, et al. The paradigm change from reactive medical services to 3PM in ischemic stroke: a holistic approach utilising tear fluid multi-omics, mitochondria as a vital biosensor and AI-based multi-professional data interpretation. EPMA J. 2024;15:1–23. 10.1007/s13167-024-00356-6. 38463624 10.1007/s13167-024-00356-6 PMC10923756

[8] Golubnitschaja O, Sargheini N, Bastert J. Mitochondria in cutaneous health, disease, ageing and rejuvenation—the 3PM-guided mitochondria-centric dermatology. EPMA J. 2025;16:1–15. 10.1007/s13167-025-00400-z. 39991093 10.1007/s13167-025-00400-z PMC11842662

[9] Koklesova L, Mazurakova A, Samec M, Kudela E, Biringer K, Kubatka P, et al. Mitochondrial health quality control: measurements and interpretation in the framework of predictive, preventive, and personalized medicine. EPMA J. 2022;13:177–93. 10.1007/s13167-022-00281-6. 35578648 10.1007/s13167-022-00281-6 PMC9096339

[10] Pesta M, Mrazova B, Kapalla M, Kulda V, Gkika E, Golubnitschaja O. Mitochondria-based holistic 3PM approach as the game-changer for individualised rehabilitation-the proof-of-principle model by treated breast cancer survivors. EPMA J. 2024;15:559–71. 10.1007/s13167-024-00386-0. 39635015 10.1007/s13167-024-00386-0 PMC11612048

[11] Lejri I, Grimm A, Eckert A. Mitochondria, estrogen and female brain aging. Front Aging Neurosci. 2018;10:124. 10.3389/fnagi.2018.00124. 29755342 10.3389/fnagi.2018.00124 PMC5934418

[12] Picard M, Wallace DC, Burelle Y. The rise of mitochondria in medicine. Mitochondrion. 2016;30:105–16. 10.1016/j.mito.2016.07.003. 27423788 10.1016/j.mito.2016.07.003 PMC5023480

[13] Zhang X, Gao Y, zhang S, Wang Y, Pei X, Chen Y, et al. Mitochondrial dysfunction in the regulation of aging and aging-related diseases. Cell Commun Signal. 2025;23:290. 10.1186/s12964-025-02308-7. 40537801 10.1186/s12964-025-02308-7 PMC12177975

[14] Vryonidou A, Paschou SA, Muscogiuri G, Orio F, Goulis DG. Mechanisms in endocrinology: metabolic syndrome through the female life cycle. Eur J Endocrinol. 2015;173:R153–63. 10.1530/EJE-15-0275. 26034072 10.1530/EJE-15-0275

[15] Boisseau N, Isacco L. Substrate metabolism during exercise: sexual dimorphism and women’s specificities. Eur J Sport Sci. 2022;22:672–83. 10.1080/17461391.2021.1943713. 34134602 10.1080/17461391.2021.1943713

[16] LöFberg IE, Karppinen JE, Laatikainen-Raussi V, Lehti M, Hackney AC, Ihalainen JK, et al. Resting energy expenditure, metabolic and sex hormones in two phases of the menstrual and hormonal contraceptive cycles. Med Sci Sports Exerc. 2024;56:2285–95. 10.1249/MSS.0000000000003518. 39086066 10.1249/MSS.0000000000003518

[17] Malo-Vintimilla L, Aguirre C, Vergara A, Fernández-Verdejo R, Galgani JE. Resting energy metabolism and sweet taste preference during the menstrual cycle in healthy women. Br J Nutr. 2024;131:384–90. 10.1017/S0007114523001927. 37641942 10.1017/S0007114523001927 PMC10784125

[18] Hackney AC. Menstrual cycle hormonal changes and energy substrate metabolism in exercising women: a perspective. Int J Environ Res Public Health. 2021;18:10024. 10.3390/ijerph181910024. 34639326 10.3390/ijerph181910024 PMC8508274

[19] Ko S-H, Jung Y. Energy metabolism changes and dysregulated lipid metabolism in postmenopausal women. Nutrients. 2021;13:4556. 10.3390/nu13124556. 34960109 10.3390/nu13124556 PMC8704126

[20] Ko S-H, Kim H-S. Menopause-associated lipid metabolic disorders and foods beneficial for postmenopausal women. Nutrients. 2020;12:202. 10.3390/nu12010202. 31941004 10.3390/nu12010202 PMC7019719

[21] Goncharenko V, Bubnov R, Polivka J, Zubor P, Biringer K, Bielik T, et al. Vaginal dryness: individualised patient profiles, risks and mitigating measures. EPMA J. 2019;10:73–9. 10.1007/s13167-019-00164-3. 30984316 10.1007/s13167-019-00164-3 PMC6459457

[22] Martuliak I, Golubnitschaja O, Chvala L, Kapalla M, Ferencik M, Bubeliny M, et al. Pain chronification risk assessment: advanced phenotyping and scoring for prediction and treatments tailored to individualized patient profile. EPMA J. 2024;15:739–50. 10.1007/s13167-024-00383-3. 39635026 10.1007/s13167-024-00383-3 PMC11612039

[23] Wang Y, Diaz Brinton R. Estrogen regulation of mitochondrial respiration is cell type and ER subtype specific. Alzheimers Dement. 2017;13:P665-6. 10.1016/j.jalz.2017.06.801.

[24] Klinge CM. Estrogenic control of mitochondrial function. Redox Biol. 2020;31:101435. 10.1016/j.redox.2020.101435. 32001259 10.1016/j.redox.2020.101435 PMC7212490

[25] Irwin RW, Yao J, Hamilton RT, Cadenas E, Brinton RD, Nilsen J. Progesterone and estrogen regulate oxidative metabolism in brain mitochondria. Endocrinology. 2008;149:3167–75. 10.1210/en.2007-1227. 18292191 10.1210/en.2007-1227 PMC2408802

[26] Beikoghli Kalkhoran S, Kararigas G. Oestrogenic regulation of mitochondrial dynamics. Int J Mol Sci. 2022;23:1118. 10.3390/ijms23031118. 35163044 10.3390/ijms23031118 PMC8834780

[27] Torrens-Mas M, Pons D-G, Sastre-Serra J, Oliver J, Roca P. Sexual hormones regulate the redox status and mitochondrial function in the brain. Pathological implications. Redox Biol. 2020;31:101505. 10.1016/j.redox.2020.101505. 32201220 10.1016/j.redox.2020.101505 PMC7212485

[28] Lynch S, Boyett JE, Smith MR, Giordano-Mooga S. Sex hormone regulation of proteins modulating mitochondrial metabolism, dynamics and inter-organellar cross talk in cardiovascular disease. Front Cell Dev Biol. 2021;8:610516. 10.3389/fcell.2020.610516. 33644031 10.3389/fcell.2020.610516 PMC7905018

[29] Shaia KL, Harris BS, Selter JH, Price TM. Reproductive functions of the mitochondrial progesterone receptor (PR-M). Reprod Sci. 2023;30:1443–52. 10.1007/s43032-022-01092-w. 36255658 10.1007/s43032-022-01092-w

[30] Yoh K, Ikeda K, Horie K, Inoue S. Roles of estrogen, estrogen receptors, and estrogen-related receptors in skeletal muscle: regulation of mitochondrial function. Int J Mol Sci. 2023;24:1853. 10.3390/ijms24031853. 36768177 10.3390/ijms24031853 PMC9916347

[31] Shaw GA, Hyer MM, Dustin E, Dyer SK, Targett IL, Neigh GN. Acute LPS exposure increases synaptosomal metabolism during estrus but not diestrus. Physiol Behav. 2021;239:113523. 10.1016/j.physbeh.2021.113523. 34229031 10.1016/j.physbeh.2021.113523

[32] Fliegner D, Ellieva A, Angelov A, Petrov G, Regitz-Zagrosek V. Sex differences and estrogen effects in cardiac mitochondria in human aortic stenosis and in the mouse heart. Front Endocrinol. 2023;14:1181044. 10.3389/fendo.2023.1181044. 10.3389/fendo.2023.1181044 PMC10617023 37916152

[33] Wang T, Mao Z, Chen S, Shang Y, McLean JW, Stanton JB, et al. Key role of ERβ in estrogenic regulation of neuronal mitochondrial function. Alzheimer’s & Dementia. 2024;20:e086633. 10.1002/alz.086633.

[34] Yokota T. Skeletal muscle mitochondria: a potential target for postmenopausal hormone replacement therapy. Acta Physiol. 2024;240:e14149. 10.1111/apha.14149. 10.1111/apha.14149 38629467

[35] Rattanasopa C, Phungphong S, Wattanapermpool J, Bupha-Intr T. Significant role of estrogen in maintaining cardiac mitochondrial functions. J Steroid Biochem Mol Biol. 2015;147:1–9. 10.1016/j.jsbmb.2014.11.009. 25448746 10.1016/j.jsbmb.2014.11.009

[36] Rodríguez-Cuenca S, Monjo M, Gianotti M, Proenza AM, Roca P. Expression of mitochondrial biogenesis-signaling factors in brown adipocytes is influenced specifically by 17β-estradiol, testosterone, and progesterone. Am J Physiol Endocrinol Metab. 2007;292:E340-6. 10.1152/ajpendo.00175.2006. 16954335 10.1152/ajpendo.00175.2006

[37] Torres MJ, Kew KA, Ryan TE, Pennington ER, Lin C-T, Buddo KA, et al. 17β-Estradiol directly lowers mitochondrial membrane microviscosity and improves bioenergetic function in skeletal muscle. Cell Metab. 2018;27:167–e797. 10.1016/j.cmet.2017.10.003. 29103922 10.1016/j.cmet.2017.10.003 PMC5762397

[38] Marotta A. Mitochondrial and redox dysfunction in post-menopause as risk factor of neurodegenerative disease: a pilot study testing the role of a validated Japanese functional food. J Biol Regul Homeost Agents. 2020;34:111–21. 10.23812/19-315-A. 32148012 10.23812/19-315-A

[39] Lopes FB, Sarandy MM, Novaes RD, Valacchi G, Gonçalves RV. Oxinflammatory responses in the wound healing process: a systematic review. Antioxidants. 2024;13:823. 10.3390/antiox13070823. 39061892 10.3390/antiox13070823 PMC11274091

[40] Cieślak M, Wojtczak A, Cieślak M. Role of pro-inflammatory cytokines of pancreatic islets and prospects of elaboration of new methods for the diabetes treatment. Acta Biochim Pol. 2015;62:15–21. 10.18388/abp.2014_853. 25781159 10.18388/abp.2014_853

[41] Makhezer N, Ben Khemis M, Liu D, Khichane Y, Marzaioli V, Tlili A, et al. NOX1-derived ROS drive the expression of Lipocalin-2 in colonic epithelial cells in inflammatory conditions. Mucosal Immunol. 2019;12:117–31. 10.1038/s41385-018-0086-4. 30279516 10.1038/s41385-018-0086-4

[42] Zhang J, Wang X, Vikash V, Ye Q, Wu D, Liu Y, et al. ROS and ROS-mediated cellular signaling. Oxid Med Cell Longev. 2016;2016:4350965. 10.1155/2016/4350965. 26998193 10.1155/2016/4350965 PMC4779832

[43] Schieber M, Chandel NS. ROS function in redox signaling and oxidative stress. Curr Biol. 2014;24:R453–62. 10.1016/j.cub.2014.03.034. 24845678 10.1016/j.cub.2014.03.034 PMC4055301

[44] Juan CA, de la Pérez Lastra JM, Plou FJ, Pérez-Lebeña E. The chemistry of reactive oxygen species (ROS) revisited: outlining their role in biological macromolecules (DNA, lipids and proteins) and induced pathologies. Int J Mol Sci. 2021;22:4642. 10.3390/ijms22094642. 33924958 10.3390/ijms22094642 PMC8125527

[45] Rowe LA, Degtyareva N, Doetsch PW. DNA damage-induced reactive oxygen species (ROS) stress response in *Saccharomyces cerevisiae*. Free Radic Biol Med. 2008;45:1167–77. 10.1016/j.freeradbiomed.2008.07.018. 18708137 10.1016/j.freeradbiomed.2008.07.018 PMC2643028

[46] Greenlee-Wacker MC, Nauseef WM. IFN-γ targets macrophage-mediated immune responses toward *Staphylococcus aureus*. J Leukoc Biol. 2017;101:751–8. 10.1189/jlb.4A1215-565RR. 27707882 10.1189/jlb.4A1215-565RR PMC5295848

[47] Hsieh LT-H, Frey H, Nastase M-V, Tredup C, Hoffmann A, Poluzzi C, et al. Bimodal role of NADPH oxidases in the regulation of biglycan-triggered IL-1β synthesis. Matrix Biol. 2016;49:61–81. 10.1016/j.matbio.2015.12.005. 26689330 10.1016/j.matbio.2015.12.005 PMC6953411

[48] Han Y, Zhang Y-Y, Pan Y-Q, Zheng X-J, Liao K, Mo H-Y, et al. IL-1β-associated NNT acetylation orchestrates iron-sulfur cluster maintenance and cancer immunotherapy resistance. Mol Cell. 2023;83:1887–e9028. 10.1016/j.molcel.2023.05.011. 37244254 10.1016/j.molcel.2023.05.011

[49] Lee J, Lee S, Min S, Kang SW. RIP3-dependent accumulation of mitochondrial superoxide anions in TNF-α-induced necroptosis. Mol Cells. 2022;45:193–201. 10.14348/molcells.2021.0260. 35289306 10.14348/molcells.2021.0260 PMC9001146

[50] Kim JJ, Lee SB, Park JK, Yoo YD. TNF-α-induced ROS production triggering apoptosis is directly linked to Romo1 and Bcl-XL. Cell Death Differ. 2010;17:1420–34. 10.1038/cdd.2010.19. 20203691 10.1038/cdd.2010.19

[51] Roberge S, Roussel J, Andersson DC, Meli AC, Vidal B, Blandel F, et al. TNF-α-mediated caspase-8 activation induces ROS production and TRPM2 activation in adult ventricular myocytes. Cardiovasc Res. 2014;103:90–9. 10.1093/cvr/cvu112. 24802330 10.1093/cvr/cvu112

[52] Kastl L, Sauer SW, Ruppert T, Beissbarth T, Becker MS, Süss D, et al. TNF-α mediates mitochondrial uncoupling and enhances ROS‐dependent cell migration *via* NF‐κB activation in liver cells. FEBS Lett. 2014;588:175–83. 10.1016/j.febslet.2013.11.033. 24316229 10.1016/j.febslet.2013.11.033

[53] Sawada M, Kiyono T, Nakashima S, Shinoda J, Naganawa T, Hara S, et al. Molecular mechanisms of TNF-α-induced ceramide formation in human glioma cells: P53-mediated oxidant stress-dependent and -independent pathways. Cell Death Differ. 2004;11:997–1008. 10.1038/sj.cdd.4401438. 15131591 10.1038/sj.cdd.4401438

[54] Michalska M, Wolf G, Walther R, Newsholme P. <article-title update="added">Effects of pharmacological inhibition of NADPH oxidase or iNOS on pro-inflammatory cytokine, palmitic acid or H2O2-induced mouse islet or clonal pancreatic β-cell dysfunction. Biosci Rep. 2010;30:445–53. 10.1042/BSR20090138. 20178457 10.1042/BSR20090138

[55] Subasinghe W, Syed I, Kowluru A. Phagocyte-like NADPH oxidase promotes cytokine-induced mitochondrial dysfunction in pancreatic β-cells: evidence for regulation by Rac1. Am J Physiol Regul Integr Comp Physiol. 2011;300:R12-20. 10.1152/ajpregu.00421.2010. 20943855 10.1152/ajpregu.00421.2010 PMC3023281

[56] Casazza K, Hanks LJ, Alvarez JA. Role of various cytokines and growth factors in pubertal development. In: Jürimäe J, Hills AP, Jürimäe T, editors. Med Sport Sci. S. Karger AG;2010. pp. 14–31. https://karger.com/chapter/doi/10.1159/000321969 10.1159/000321969 20956857

[57] Rogol AD. Growth at puberty: interaction of androgens and growth hormone. Med Sci Sports Exerc. 1994;26:767–70. 10.1249/00005768-199406000-00017. 7914344 10.1249/00005768-199406000-00017

[58] Noirrit-Esclassan E, Valera M-C, Tremollieres F, Arnal J-F, Lenfant F, Fontaine C, et al. Critical role of estrogens on bone homeostasis in both male and female: from physiology to medical implications. Int J Mol Sci. 2021;22:1568. 10.3390/ijms22041568. 33557249 10.3390/ijms22041568 PMC7913980

[59] Sciarra F, Campolo F, Franceschini E, Carlomagno F, Venneri M. Gender-specific impact of sex hormones on the immune system. Int J Mol Sci. 2023;24:6302. 10.3390/ijms24076302. 37047274 10.3390/ijms24076302 PMC10094624

[60] Leung K-C, Johannsson G, Leong GM, Ho KKY. Estrogen regulation of growth hormone action. Endocr Rev. 2004;25:693–721. 10.1210/er.2003-0035. 15466938 10.1210/er.2003-0035

[61] Christoforidis A, Maniadaki I, Stanhope R. Growth hormone / insulin-like growth factor-1 axis during puberty. Pediatr Endocrinol Rev. 2005;3:5–10. 16369208

[62] Zhunina OA, Yabbarov NG, Grechko AV, Starodubova AV, Ivanova E, Nikiforov NG, et al. The role of mitochondrial dysfunction in vascular disease, tumorigenesis, and diabetes. Front Mol Biosci. 2021;8:671908. 10.3389/fmolb.2021.671908. 34026846 10.3389/fmolb.2021.671908 PMC8138126

[63] Hertiš Petek T, Petek T, Močnik M, Marčun Varda N. Systemic inflammation, oxidative stress and cardiovascular health in children and adolescents: a systematic review. Antioxidants. 2022;11:894. 10.3390/antiox11050894. 35624760 10.3390/antiox11050894 PMC9137597

[64] Rizwan H, Pal S, Sabnam S, Pal A. High glucose augments ROS generation regulates mitochondrial dysfunction and apoptosis via stress signalling cascades in keratinocytes. Life Sci. 2020;241:117148. 10.1016/j.lfs.2019.117148. 31830478 10.1016/j.lfs.2019.117148

[65] Barrera M-J, Aguilera S, Castro I, Carvajal P, Jara D, Molina C, et al. Dysfunctional mitochondria as critical players in the inflammation of autoimmune diseases: potential role in Sjögren’s syndrome. Autoimmun Rev. 2021;20:102867. 10.1016/j.autrev.2021.102867. 34118452 10.1016/j.autrev.2021.102867

[66] Forrester SJ, Kikuchi DS, Hernandes MS, Xu Q, Griendling KK. Reactive oxygen species in metabolic and inflammatory signaling. Circ Res. 2018;122:877–902. 10.1161/CIRCRESAHA.117.311401. 29700084 10.1161/CIRCRESAHA.117.311401 PMC5926825

[67] Patergnani S, Bouhamida E, Leo S, Pinton P, Rimessi A. Mitochondrial oxidative stress and “mito-inflammation”: actors in the diseases. Biomedicines. 2021;9:216. 10.3390/biomedicines9020216. 33672477 10.3390/biomedicines9020216 PMC7923430

[68] Nesci S, Spagnoletta A, Oppedisano F. Inflammation, mitochondria and natural compounds together in the circle of trust. Int J Mol Sci. 2023;24:6106. 10.3390/ijms24076106. 37047080 10.3390/ijms24076106 PMC10094238

[69] Sanghavi M, Rutherford JD. Cardiovascular physiology of pregnancy. Circulation. 2014;130:1003–8. 10.1161/CIRCULATIONAHA.114.009029. 25223771 10.1161/CIRCULATIONAHA.114.009029

[70] Hibbard JU, Shroff SG, Cunningham FG. Cardiovascular alterations in normal and preeclamptic pregnancy. In: Chesleys Hypertens Disord Pregnancy. Elsevier; 2015. p. 291–313.

[71] Sherman-Brown A, Hameed AB. Cardiovascular disease screening in pregnancy. Clin Obstet Gynecol. 2020;63:808–14. 10.1097/GRF.0000000000000565. 33060374 10.1097/GRF.0000000000000565

[72] Eberle C, Fasig T, Brüseke F, Stichling S. Impact of maternal prenatal stress by glucocorticoids on metabolic and cardiovascular outcomes in their offspring: a systematic scoping review. PLoS One. 2021;16:e0245386. 10.1371/journal.pone.0245386. 33481865 10.1371/journal.pone.0245386 PMC7822275

[73] Gongora M, Wenger N. Cardiovascular complications of pregnancy. Int J Mol Sci. 2015;16:23905–28. 10.3390/ijms161023905. 26473833 10.3390/ijms161023905 PMC4632731

[74] Staff AC, Costa ML, Dechend R, Jacobsen DP, Sugulle M. Hypertensive disorders of pregnancy and long-term maternal cardiovascular risk: bridging epidemiological knowledge into personalized postpartum care and follow-up. Pregnancy Hypertens. 2024;36:101127. 10.1016/j.preghy.2024.101127. 38643570 10.1016/j.preghy.2024.101127

[75] Bhardwaj G, Smitha MV, Jelly P, Stephen S, Cook JE, Panda S. Breastfeeding challenges experienced by mothers following multiple births – a systematic review and meta-synthesis of quantitative, qualitative, and mixed-methods studies. Breastfeed Med. 2025;20:219–30. 10.1089/bfm.2024.0207. 39749367 10.1089/bfm.2024.0207

[76] Porta R, Capdevila E, Botet F, Ginovart G, Moliner E, Nicolàs M, et al. Breastfeeding disparities between multiples and singletons by NICU discharge. Nutrients. 2019;11:2191. 10.3390/nu11092191. 31547239 10.3390/nu11092191 PMC6770324

[77] Blauvelt CA, Turcios S, Wen T, Boscardin J, Seidman D. Breastfeeding initiation in people with hepatitis C virus infection in the united States. Obstet Gynecol. 2024;143:683–9. 10.1097/AOG.0000000000005555. 38513240 10.1097/AOG.0000000000005555

[78] Jonsdottir RB, Flacking R, Jonsdottir H. Breastfeeding initiation, duration, and experiences of mothers of late preterm twins: a mixed-methods study. Int Breastfeed J. 2022;17:68. 10.1186/s13006-022-00507-3. 36076279 10.1186/s13006-022-00507-3 PMC9461222

[79] Borges VF, Lyons TR, Germain D, Schedin P. Postpartum involution and cancer: an opportunity for targeted breast cancer prevention and treatments? Cancer Res. 2020;80:1790–8. 10.1158/0008-5472.CAN-19-3448. 32075799 10.1158/0008-5472.CAN-19-3448 PMC8285071

[80] Taylor VJ. Lactation from the inside out: maternal homeorhetic gastrointestinal adaptations regulating energy and nutrient flow into milk production. Mol Cell Endocrinol. 2023;559:111797. 10.1016/j.mce.2022.111797. 36243202 10.1016/j.mce.2022.111797

[81] Kovacs CS. Maternal mineral and bone metabolism during pregnancy, lactation, and post-weaning recovery. Physiol Rev. 2016;96:449–547. 10.1152/physrev.00027.2015. 26887676 10.1152/physrev.00027.2015

[82] Bell AW. Regulation of organic nutrient metabolism during transition from late pregnancy to early lactation. J Anim Sci. 1995;73:2804. 10.2527/1995.7392804x. 8582872 10.2527/1995.7392804x

[83] Álvarez-Delgado C. The role of mitochondria and mitochondrial hormone receptors on the bioenergetic adaptations to lactation. Mol Cell Endocrinol. 2022;551:111661. 10.1016/j.mce.2022.111661. 35483518 10.1016/j.mce.2022.111661

[84] Favorit V, Hood WR, Kavazis AN, Villamediana P, Yap KN, Parry HA, et al. Mitochondrial bioenergetics of extramammary tissues in lactating dairy cattle. Animals. 2021;11:2647. 10.3390/ani11092647. 34573613 10.3390/ani11092647 PMC8467216

[85] Favorit V, Hood WR, Kavazis AN, Skibiel AL. Graduate student literature review: mitochondrial adaptations across lactation and their molecular regulation in dairy cattle. J Dairy Sci. 2021;104:10415–25. 10.3168/jds.2021-20138. 34218917 10.3168/jds.2021-20138 PMC13317002

[86] Mowry AV, Donoviel ZS, Kavazis AN, Hood WR. Mitochondrial function and bioenergetic trade-offs during lactation in the house mouse (*Mus musculus*). Ecol Evol. 2017;7:2994–3005. 10.1002/ece3.2817. 28479999 10.1002/ece3.2817 PMC5415517

[87] Parry HA, Randall RB, Hyatt HW, Hood WR, Kavazis AN. Short and long-term effect of reproduction on mitochondrial dynamics and autophagy in rats. Heliyon. 2021;7:e08070. 10.1016/j.heliyon.2021.e08070. 34622072 10.1016/j.heliyon.2021.e08070 PMC8479403

[88] Hyatt HW, Zhang Y, Hood WR, Kavazis AN. Lactation has persistent effects on a mother’s metabolism and mitochondrial function. Sci Rep. 2017;7:17118. 10.1038/s41598-017-17418-7. 29215072 10.1038/s41598-017-17418-7 PMC5719424

[89] Liu K, Zhang L, Xu X, Song M, Ding H, Xiao L, et al. Lactational high weight loss impairs follicular development by causing mitochondrial dysfunction of ovarian cells in sows and mitigated by butyrate supplement. J Adv Res. 2025;78:11–28. 10.1016/j.jare.2025.01.050. 39892609 10.1016/j.jare.2025.01.050 PMC12684926

[90] Theys N, Ahn M-T, Bouckenooghe T, Reusens B, Remacle C. Maternal malnutrition programs pancreatic islet mitochondrial dysfunction in the adult offspring. J Nutr Biochem. 2011;22:985–94. 10.1016/j.jnutbio.2010.08.015. 21190832 10.1016/j.jnutbio.2010.08.015

[91] Lee YY, Lee H-J, Lee S-S, Koh JS, Jin CJ, Park S-H, et al. Taurine supplementation restored the changes in pancreatic islet mitochondria in the fetal protein-malnourished rat. Br J Nutr. 2011;106:1198–206. 10.1017/S0007114511001632. 21736818 10.1017/S0007114511001632

[92] Burger HG, Hale GE, Robertson DM, Dennerstein L. A review of hormonal changes during the menopausal transition: focus on findings from the Melbourne women’s midlife health project. Hum Reprod Update. 2007;13:559–65. 10.1093/humupd/dmm020. 17630397 10.1093/humupd/dmm020

[93] Monteleone P, Mascagni G, Giannini A, Genazzani AR, Simoncini T. Symptoms of menopause — global prevalence, physiology and implications. Nat Rev Endocrinol. 2018;14:199–215. 10.1038/nrendo.2017.180. 29393299 10.1038/nrendo.2017.180

[94] Santoro N, Roeca C, Peters BA, Neal-Perry G. The menopause transition: signs, symptoms, and management options. J Clin Endocrinol Metab. 2021;106:1–15. 10.1210/clinem/dgaa764. 33095879 10.1210/clinem/dgaa764

[95] Villa A, Rizzi N, Vegeto E, Ciana P, Maggi A. Estrogen accelerates the resolution of inflammation in macrophagic cells. Sci Rep. 2015;5:15224. 10.1038/srep15224. 26477569 10.1038/srep15224 PMC4609992

[96] Pernoud LE, Gardiner PA, Fraser SD, Dillon-Rossiter K, Dean MM, Schaumberg MA. A systematic review and meta-analysis investigating differences in chronic inflammation and adiposity before and after menopause. Maturitas. 2024;190:108119. 10.1016/j.maturitas.2024.108119. 39332331 10.1016/j.maturitas.2024.108119

[97] Oparil S, Xing D, Hage F, Roger White C, Giordano S. Estrogen signaling modulates mitochondrial ROS production and mitochondrial function in mouse bone marrow macrophages. Free Radic Biol Med. 2017;112:203. 10.1016/j.freeradbiomed.2017.10.320.

[98] McCarthy M, Raval AP. The peri-menopause in a woman’s life: a systemic inflammatory phase that enables later neurodegenerative disease. J Neuroinflammation. 2020;17:317. 10.1186/s12974-020-01998-9. 33097048 10.1186/s12974-020-01998-9 PMC7585188

[99] Torres-Torres J, Monroy-Muñoz IE, Perez-Duran J, Solis-Paredes JM, Camacho-Martinez ZA, Baca D, et al. Cellular and molecular pathophysiology of gestational diabetes. Int J Mol Sci. 2024;25:11641. 10.3390/ijms252111641. 39519193 10.3390/ijms252111641 PMC11546748

[100] Sferruzzi-Perri AN. Placental mitochondria central to gestational diabetes pathogenesis? J Physiol. 2021;599:1019–20. 10.1113/JP281041. 33337541 10.1113/JP281041

[101] Kolac UK, Kurek Eken M, Ünübol M, Donmez Yalcin G, Yalcin A. The effect of gestational diabetes on the expression of mitochondrial fusion proteins in placental tissue. Placenta. 2021;115:106–14. 10.1016/j.placenta.2021.09.015. 34600274 10.1016/j.placenta.2021.09.015

[102] Zhao X, Zhang W, Jiang F, Chen X, Chen C, Wang M, et al. Excessive palmitic acid disturbs macrophage α-ketoglutarate/succinate metabolism and causes adipose tissue insulin resistance associated with gestational diabetes mellitus. Free Radic Biol Med. 2024;222:424–36. 10.1016/j.freeradbiomed.2024.06.029. 38960008 10.1016/j.freeradbiomed.2024.06.029

[103] McCarthy CM, McElwain CJ, Musumeci A, McCarthy FP. Evidence of tissue specific alterations in inflammatory profiles in gestational diabetes mellitus. J Reprod Immunol. 2023;159:104000. 10.1016/j.jri.2023.104000.

[104] Newsholme P, Cruzat VF, Keane KN, Carlessi R, de Bittencourt PIH. Molecular mechanisms of ROS production and oxidative stress in diabetes. Biochem J. 2016;473:4527–50. 10.1042/BCJ20160503C. 27941030 10.1042/BCJ20160503C

[105] Joshi NP, Madiwale SD, Sundrani DP, Joshi SR. Fatty acids, inflammation and angiogenesis in women with gestational diabetes mellitus. Biochimie. 2023;212:31–40. 10.1016/j.biochi.2023.04.005. 37059350 10.1016/j.biochi.2023.04.005

[106] Ma S, Li P, Li D, Zhou M, Li L, Yin W, et al. Increasing systemic chronic inflammation mediated the association between poor sleep during pregnancy and gestational cardiovascular health. Sleep Health. 2023;9:460–6. 10.1016/j.sleh.2023.01.015. 37088599 10.1016/j.sleh.2023.01.015

[107] De Oliveira MP, da Silva LE, Fernandes BB, Steiner MR, Pistóia DG, Santos Cichella Tdos, et al. The impact of obesity on mitochondrial dysfunction during pregnancy. Mol Cell Endocrinol. 2025;598:112463. 10.1016/j.mce.2025.112463. 39832615 10.1016/j.mce.2025.112463

[108] San Juan-Reyes S, Gómez-Oliván LM, Islas-Flores H, Dublán-García O. Oxidative stress in pregnancy complicated by preeclampsia. Arch Biochem Biophys. 2020;681:108255. 10.1016/j.abb.2020.108255. 31904364 10.1016/j.abb.2020.108255

[109] Marín R, Chiarello DI, Abad C, Rojas D, Toledo F, Sobrevia L. Oxidative stress and mitochondrial dysfunction in early-onset and late-onset preeclampsia. Biochimica et Biophysica Acta (BBA) - Molecular Basis of Disease. 2020;1866:165961. 10.1016/j.bbadis.2020.165961. 32916282 10.1016/j.bbadis.2020.165961

[110] Covarrubias AE, Lecarpentier E, Lo A, Salahuddin S, Gray KJ, Karumanchi SA, et al. AP39, a modulator of mitochondrial bioenergetics, reduces antiangiogenic response and oxidative stress in hypoxia-exposed trophoblasts. Am J Pathol. 2019;189:104–14. 10.1016/j.ajpath.2018.09.007. 30315766 10.1016/j.ajpath.2018.09.007 PMC6854435

[111] Torres-Torres J, Espino-y-Sosa S, Martinez-Portilla R, Borboa-Olivares H, Estrada-Gutierrez G, Acevedo-Gallegos S, et al. A narrative review on the pathophysiology of preeclampsia. Int J Mol Sci. 2024;25:7569. 10.3390/ijms25147569. 39062815 10.3390/ijms25147569 PMC11277207

[112] Vaka VR, McMaster KM, Cunningham MW, Ibrahim T, Hazlewood R, Usry N, et al. Role of mitochondrial dysfunction and reactive oxygen species in mediating hypertension in the reduced uterine perfusion pressure rat model of preeclampsia. Hypertension. 2018;72:703–11. 10.1161/HYPERTENSIONAHA.118.11290. 30012871 10.1161/HYPERTENSIONAHA.118.11290 PMC6394841

[113] Long J, Huang Y, Wang G, Tang Z, Shan Y, Shen S, et al. Mitochondrial ROS accumulation contributes to maternal hypertension and impaired remodeling of spiral artery but not IUGR in a rat PE model caused by maternal glucocorticoid exposure. Antioxidants. 2023;12:987. 10.3390/antiox12050987. 37237853 10.3390/antiox12050987 PMC10215507

[114] Goulopoulou S, Matsumoto T, Bomfim GF, Webb RC. Toll-like receptor 9 activation: a novel mechanism linking placenta-derived mitochondrial DNA and vascular dysfunction in pre-eclampsia. Clin Sci Lond. 2012;123:429–35. 10.1042/CS20120130. 22671429 10.1042/CS20120130 PMC4004352

[115] Phoswa WN, Khaliq OP. The role of oxidative stress in hypertensive disorders of pregnancy (preeclampsia, gestational hypertension) and metabolic disorder of pregnancy (gestational diabetes mellitus). Oxid Med Cell Longev. 2021;2021:5581570. 10.1155/2021/5581570. 34194606 10.1155/2021/5581570 PMC8184326

[116] Bianchi C, Taricco E, Cardellicchio M, Mandò C, Massari M, Savasi V, et al. The role of obesity and gestational diabetes on placental size and fetal oxygenation. Placenta. 2021;103:59–63. 10.1016/j.placenta.2020.10.013. 33080447 10.1016/j.placenta.2020.10.013

[117] Farabi SS, Barbour LA, Hernandez TL. Sleep-disordered breathing in pregnancy: a developmental origin of offspring obesity? J Dev Orig Health Dis. 2021;12:237–49. 10.1017/S2040174420000355. 32425147 10.1017/S2040174420000355 PMC9951118

[118] Desoye G, Carter AM. Fetoplacental oxygen homeostasis in pregnancies with maternal diabetes mellitus and obesity. Nat Rev Endocrinol. 2022;18:593–607. 10.1038/s41574-022-00717-z. 35902735 10.1038/s41574-022-00717-z

[119] Joo EH, Kim YR, Kim N, Jung JE, Han SH, Cho HY. Effect of endogenic and exogenic oxidative stress triggers on adverse pregnancy outcomes: preeclampsia, fetal growth restriction, gestational diabetes mellitus and preterm birth. Int J Mol Sci. 2021;22:10122. 10.3390/ijms221810122. 34576285 10.3390/ijms221810122 PMC8468091

[120] Holland O, Dekker Nitert M, Gallo LA, Vejzovic M, Fisher JJ, Perkins AV. Review: placental mitochondrial function and structure in gestational disorders. Placenta. 2017;54:2–9. 10.1016/j.placenta.2016.12.012. 28024805 10.1016/j.placenta.2016.12.012

[121] Smith KLM, Swiderska A, Lock MC, Graham L, Iswari W, Choudhary T, et al. Chronic developmental hypoxia alters mitochondrial oxidative capacity and reactive oxygen species production in the fetal rat heart in a sex-dependent manner. J Pineal Res. 2022;73:e12821. 10.1111/jpi.12821. 35941749 10.1111/jpi.12821 PMC9540814

[122] Azziz R, Carmina E, Chen Z, Dunaif A, Laven JSE, Legro RS, et al. Polycystic ovary syndrome. Nat Rev Dis Primers. 2016;2:16057. 10.1038/nrdp.2016.57. 27510637 10.1038/nrdp.2016.57

[123] Joham AE, Norman RJ, Stener-Victorin E, Legro RS, Franks S, Moran LJ, et al. Polycystic ovary syndrome. Lancet Diabetes Endocrinol. 2022;10:668–80. 10.1016/S2213-8587(22)00163-2. 35934017 10.1016/S2213-8587(22)00163-2

[124] Azziz R. Polycystic ovary syndrome. Obstet Gynecol. 2018;132:321–36. 10.1097/AOG.0000000000002698. 29995717 10.1097/AOG.0000000000002698

[125] Rostamtabar M, Esmaeilzadeh S, Tourani M, Rahmani A, Baee M, Shirafkan F, et al. Pathophysiological roles of chronic low-grade inflammation mediators in polycystic ovary syndrome. J Cell Physiol. 2021;236:824–38. 10.1002/jcp.29912. 32617971 10.1002/jcp.29912

[126] Kobayashi H, Matsubara S, Yoshimoto C, Shigetomi H, Imanaka S. A comprehensive review of the contribution of mitochondrial DNA mutations and dysfunction in polycystic ovary syndrome, supported by secondary database analysis. Int J Mol Sci. 2025;26:1172. 10.3390/ijms26031172. 39940939 10.3390/ijms26031172 PMC11818232

[127] Dabravolski SA, Nikiforov NG, Eid AH, Nedosugova LV, Starodubova AV, Popkova TV, et al. Mitochondrial dysfunction and chronic inflammation in polycystic ovary syndrome. Int J Mol Sci. 2021;22:3923. 10.3390/ijms22083923. 33920227 10.3390/ijms22083923 PMC8070512

[128] Zhang J, Bao Y, Zhou X, Zheng L. Polycystic ovary syndrome and mitochondrial dysfunction. Reprod Biol Endocrinol. 2019;17:67. 10.1186/s12958-019-0509-4. 31420039 10.1186/s12958-019-0509-4 PMC6698037

[129] Zeng X, Huang Q, Long S, Zhong Q, Mo Z. Mitochondrial dysfunction in polycystic ovary syndrome. DNA Cell Biol. 2020;39:1401–9. 10.1089/dna.2019.5172. 32077751 10.1089/dna.2019.5172

[130] Gao Y, Zou Y, Wu G, Zheng L. Oxidative stress and mitochondrial dysfunction of granulosa cells in polycystic ovarian syndrome. Front Med. 2023;10:1193749. 10.3389/fmed.2023.1193749. 10.3389/fmed.2023.1193749 PMC10336225 37448805

[131] Zeber-Lubecka N, Ciebiera M, Hennig EE. Polycystic ovary syndrome and oxidative stress – from bench to bedside. Int J Mol Sci. 2023;24:14126. 10.3390/ijms241814126. 37762427 10.3390/ijms241814126 PMC10531631

[132] Wang Y, Yang Q, Wang H, Zhu J, Cong L, Li H, et al. NAD+ deficiency and mitochondrial dysfunction in granulosa cells of women with polycystic ovary syndrome. Biol Reprod. 2021;105:371–80. 10.1093/biolre/ioab078. 34056649 10.1093/biolre/ioab078

[133] Zhang Q, Ren J, Wang F, Pan M, Cui L, Li M, et al. Mitochondrial and glucose metabolic dysfunctions in granulosa cells induce impaired oocytes of polycystic ovary syndrome through Sirtuin 3. Free Radic Biol Med. 2022;187:1–16. 10.1016/j.freeradbiomed.2022.05.010. 35594990 10.1016/j.freeradbiomed.2022.05.010

[134] Palleja A, Mikkelsen KH, Forslund SK, Kashani A, Allin KH, Nielsen T, et al. Recovery of gut microbiota of healthy adults following antibiotic exposure. Nat Microbiol. 2018;3:1255–65. 10.1038/s41564-018-0257-9. 30349083 10.1038/s41564-018-0257-9

[135] Schwartz DJ, Langdon AE, Dantas G. Understanding the impact of antibiotic perturbation on the human microbiome. Genome Med. 2020;12:82. 10.1186/s13073-020-00782-x. 32988391 10.1186/s13073-020-00782-x PMC7523053

[136] Konstantinidis T, Tsigalou C, Karvelas A, Stavropoulou E, Voidarou C, Bezirtzoglou E. Effects of antibiotics upon the gut microbiome: a review of the literature. Biomedicines. 2020;8:502. 10.3390/biomedicines8110502. 33207631 10.3390/biomedicines8110502 PMC7696078

[137] Nel Van Zyl K, Matukane SR, Hamman BL, Whitelaw AC, Newton-Foot M. Effect of antibiotics on the human microbiome: a systematic review. Int J Antimicrob Agents. 2022;59:106502. 10.1016/j.ijantimicag.2021.106502. 34929293 10.1016/j.ijantimicag.2021.106502

[138] Fishbein SRS, Mahmud B, Dantas G. Antibiotic perturbations to the gut microbiome. Nat Rev Microbiol. 2023;21:772–88. 10.1038/s41579-023-00933-y. 37491458 10.1038/s41579-023-00933-y PMC12087466

[139] Agus A, Clément K, Sokol H. Gut microbiota-derived metabolites as central regulators in metabolic disorders. Gut. 2021;70:1174–82. 10.1136/gutjnl-2020-323071. 33272977 10.1136/gutjnl-2020-323071 PMC8108286

[140] Lin K, Zhu L, Yang L. Gut and obesity/metabolic disease: focus on microbiota metabolites. MedComm. 2022;3:e171. 10.1002/mco2.171. 36092861 10.1002/mco2.171 PMC9437302

[141] Peredo-Lovillo A, Romero-Luna HE, Jiménez-Fernández M. Health promoting microbial metabolites produced by gut microbiota after prebiotics metabolism. Food Res Int. 2020;136:109473. 10.1016/j.foodres.2020.109473. 32846558 10.1016/j.foodres.2020.109473

[142] Oliphant K, Allen-Vercoe E. Macronutrient metabolism by the human gut microbiome: major fermentation by-products and their impact on host health. Microbiome. 2019;7:91. 10.1186/s40168-019-0704-8. 31196177 10.1186/s40168-019-0704-8 PMC6567490

[143] Zhang H, Xie Y, Cao F, Song X. Gut microbiota-derived fatty acid and sterol metabolites: biotransformation and immunomodulatory functions. Gut Microbes. 2024;16:2382336. 10.1080/19490976.2024.2382336. 39046079 10.1080/19490976.2024.2382336 PMC11271093

[144] Dominic A, Hamilton D, Abe J-I. Mitochondria and chronic effects of cancer therapeutics: the clinical implications. J Thromb Thrombolysis. 2021;51:884–9. 10.1007/s11239-020-02313-2. 33079380 10.1007/s11239-020-02313-2 PMC8055726

[145] Gorini S, De Angelis A, Berrino L, Malara N, Rosano G, Ferraro E. Chemotherapeutic drugs and mitochondrial dysfunction: focus on doxorubicin, trastuzumab, and sunitinib. Oxid Med Cell Longev. 2018;2018:7582730. 10.1155/2018/7582730. 29743983 10.1155/2018/7582730 PMC5878876

[146] Golubnitschaja O. How to use an extensive flammer syndrome phenotyping for a holistic protection against health-to-disease transition – facts and practical recommendations. EPMA J. 2025;16:535–9. 10.1007/s13167-025-00423-6. 40948987 10.1007/s13167-025-00423-6 PMC12422999

[147] Smokovski I, Steinle N, Behnke A, Bhaskar SMM, Grech G, Richter K, et al. Digital biomarkers: 3PM approach revolutionizing chronic disease management - EPMA 2024 position. EPMA J. 2024;15:149–62. 10.1007/s13167-024-00364-6. 38841615 10.1007/s13167-024-00364-6 PMC11147994

[148] Kubatka P, Huniadi M, Kapinova A, Nosalova N, Varghese E, Blahutova D, et al. Flavonoid-modulated JAK-STAT signaling mitigates malignant transformation and drug resistance in breast tumors: A clinically relevant 3PM-guided innovation. J Adv Res. 2025. 10.1016/j.jare.2025.10.067.41205803 10.1016/j.jare.2025.10.067

[149] Golubnitschaja O. Mitochondrial biosensorics check-up is crucial for physical fitness and exercise intervention quality – facts and practical recommendations. Clin Bioenerg. 2025;1:11. 10.3390/clinbioenerg1020011.

[150] Zhang C, Yang Y, Chen R, Wei Y, Feng Y, Zheng W, et al. Aberrant expression of oxidative stress related proteins affects the pregnancy outcome of gestational diabetes mellitus patients. Am J Transl Res. 2019;11:269–79. 30787985 PMC6357329

[151] Li H, Yin Q, Li N, Ouyang Z, Zhong M. Plasma markers of oxidative stress in patients with gestational diabetes mellitus in the second and third trimester. Obstet Gynecol Int. 2016;2016:3865454. 10.1155/2016/3865454. 27803713 10.1155/2016/3865454 PMC5075618

[152] Ruiz ML, Gomez-Diaz RA, Leticia Valdez Gonzalez A, Ángeles Mejía S, Diaz-Flores M, Saldaña Espinoza RC, et al. Association of oxidative stress markers with incident hyperglycemia in gestational diabetes mellitus – impact of a diabetes prevention program. Diabetes. 2024;73(Supplement1):1248–P. 10.2337/db24-1248-P. 10.3390/nu17040680 PMC11858598 40005008

[153] Zovari F, Parsian H, Bijani A, Moslemnezhad A, Shirzad A. Evaluation of salivary and serum total antioxidant capacity and lipid peroxidation in postmenopausal women. Int J Dent. 2020;2020:1–5. 10.1155/2020/8860467. 10.1155/2020/8860467 PMC7685808 33281901

[154] Muthulakshmi C, Krishnan SNRSVV, Kumar R. Evaluation of salivary malondialdehyde levels to assess oxidative stress in postmenopausal women. Endocrinol Res Pract. 2024;28:211–5. 10.5152/erp.2024.444.

[155] Ogunro PS, Bolarinde AA, Owa OO, Salawu AA, Oshodi AA. Antioxidant status and reproductive hormones in women during reproductive, perimenopausal and postmenopausal phase of life. Afr J Med Med Sci. 2014;43:49–57. 25335378

[156] Unfer TC, Figueiredo CG, Zanchi MM, Maurer LH, Kemerich DM, Duarte MMF, et al. Estrogen plus progestin increase superoxide dismutase and total antioxidant capacity in postmenopausal women. Climacteric J Int Menopause Soc. 2015;18:379–88. 10.3109/13697137.2014.964669. 10.3109/13697137.2014.964669 25236970

[157] Köksal T, Yalçin SS, Uçartürk SA. Oxidant-antioxidant balance in girls with precocious puberty: a case-control study. Int J Environ Health Res. 2023;33:299–306. 10.1080/09603123.2022.2025767. 35000523 10.1080/09603123.2022.2025767

[158] Reese AT, Cho EH, Klitzman B, Nichols SP, Wisniewski NA, Villa MM, et al. Antibiotic-induced changes in the microbiota disrupt redox dynamics in the gut. eLife. 2018. 10.7554/eLife.35987. 29916366 10.7554/eLife.35987 PMC6008055

[159] Kalghatgi S, Spina CS, Costello JC, Liesa M, Morones-Ramirez JR, Slomovic S, et al. Bactericidal antibiotics induce mitochondrial dysfunction and oxidative damage in mammalian cells. Sci Transl Med. 2013;5:192ra85. 10.1126/scitranslmed.3006055. 23825301 10.1126/scitranslmed.3006055 PMC3760005

[160] Kropp M, De Clerck E, Vo T-TKS, Thumann G, Costigliola V, Golubnitschaja O. Short communication: unique metabolic signature of proliferative retinopathy in the tear fluid of diabetic patients with comorbidities – preliminary data for PPPM validation. EPMA J. 2023;14:43–51. 10.1007/s13167-023-00318-4. 36845280 10.1007/s13167-023-00318-4 PMC9944425

[161] Golubnitschaja O (2023) What is the routine mitochondrial health check-up good for? A holistic approach in the framework of 3P medicine. In: Podbielska H, Kapalla M, editors Predict prev Pers med bench bedside. Cham: Springer International Publishing, pp. 19–44. 10.1007/978-3-031-34884-6_3

[162] 3P Medicon your risk reducer. [Internet]. 3P Medicon; [cited 2025 Nov 14]. https://www.3pmedicon.com/en/scientific-evidence/compromised-mitochondrial-health. Accessed 14 Nov 2025.

[163] Luo Q, Zhou J, Wu H, Qiu X, Xian C, Zhan X, et al. Associations between peripheral blood mitochondrial genomic variants and gestational diabetes mellitus and postpartum abnormal glucose metabolism. J Diabetes Investig. 2025;16:2092–100. 10.1111/jdi.70152. 40937888 10.1111/jdi.70152 PMC12578659

[164] Akbari M, Masjedi F, Nekooeian M, Dara M, Roozbeh J. Circulating cell-free mitochondrial DNA in diabetes mellitus: current insights and unexplored frontiers. J Diabetes Investig. 2025;16:1575–82. 10.1111/jdi.70071. 40504099 10.1111/jdi.70071 PMC12400358

[165] Sahu DK, Abraham J. Plasma mitochondrial DNA is elevated in maternal serum at first trimester and may serve as a biomarker for prediction of gestational diabetes mellitus. J Diabetes. 2023;15:1095–102. 10.1111/1753-0407.13462. 37658630 10.1111/1753-0407.13462 PMC10755614

[166] Pandey D, Yevale A, Naha R, Kuthethur R, Chakrabarty S, Satyamoorthy K. Mitochondrial DNA copy number variation – a potential biomarker for early onset preeclampsia. Pregnancy Hypertens. 2021;23:1–4. 10.1016/j.preghy.2020.10.002. 33160129 10.1016/j.preghy.2020.10.002

[167] Qiu C, Hevner K, Enquobahrie DA, Williams MA. A case-control study of maternal blood mitochondrial DNA copy number and preeclampsia risk. Int J Mol Epidemiol Genet. 2012;3:237–44. 23050054 PMC3459217

[168] Cushen SC, Ricci CA, Bradshaw JL, Silzer T, Blessing A, Sun J, et al. Reduced maternal Circulating cell-free mitochondrial DNA is associated with the development of preeclampsia. J Am Heart Assoc. 2022;11:e021726. 10.1161/JAHA.121.021726. 35014857 10.1161/JAHA.121.021726 PMC9238514

[169] Marschalek J, Wohlrab P, Ott J, Wojta J, Speidl W, Klein KU, et al. Maternal serum mitochondrial DNA (mtDNA) levels are elevated in preeclampsia - A matched case-control study. Pregnancy Hypertens. 2018;14:195–9. 10.1016/j.preghy.2018.10.003. 30527111 10.1016/j.preghy.2018.10.003

[170] Von Holle A, Adami H-O, Baglietto L, Berrington de Gonzalez A, Bertrand KA, Blot W, et al. BMI and breast cancer risk around age at menopause. Cancer Epidemiol. 2024;89:102545. 10.1016/j.canep.2024.102545. 38377945 10.1016/j.canep.2024.102545 PMC10942753

[171] Golubnitschaja O. Mitochondrion: the subordinated partner who agreed to come short but insists in healthy life. In: Wang W, editor. Suboptimal health. Cham: Springer Nature Switzerland; 2024. pp. 17–29. https://link.springer.com/. 10.1007/978-3-031-46891-9_3.

[172] Lin Y, Wang Y, Li P, PPARα. An emerging target of metabolic syndrome, neurodegenerative and cardiovascular diseases. Front Endocrinol. 2022;13:1074911. 10.3389/fendo.2022.1074911. 10.3389/fendo.2022.1074911 PMC9800994 36589809

[173] Boden G, Homko C, Mozzoli M, Showe LC, Nichols C, Cheung P. Thiazolidinediones upregulate fatty acid uptake and oxidation in adipose tissue of diabetic patients. Diabetes. 2005;54:880–5. 10.2337/diabetes.54.3.880. 15734868 10.2337/diabetes.54.3.880

[174] Xi Y, Zhang Y, Zhu S, Luo Y, Xu P, Huang Z. PPAR-mediated toxicology and applied Pharmacology. Cells. 2020;9:352. 10.3390/cells9020352. 32028670 10.3390/cells9020352 PMC7072218

[175] Zhan X, Li J, Guo Y, Golubnitschaja O. Mass spectrometry analysis of human tear fluid biomarkers specific for ocular and systemic diseases in the context of 3P medicine. EPMA J. 2021;12:449–75. 10.1007/s13167-021-00265-y. 34876936 10.1007/s13167-021-00265-y PMC8639411

